# Bacterial Hsp70 resolves misfolded states and accelerates productive folding of a multi-domain protein

**DOI:** 10.1038/s41467-019-14245-4

**Published:** 2020-01-17

**Authors:** Rahmi Imamoglu, David Balchin, Manajit Hayer-Hartl, F. Ulrich Hartl

**Affiliations:** Max Planck Institute of Biochemistry, Department of Cellular Biochemistry, Martinsried, Germany

**Keywords:** Enzyme mechanisms, Protein folding

## Abstract

The ATP-dependent Hsp70 chaperones (DnaK in *E. coli*) mediate protein folding in cooperation with J proteins and nucleotide exchange factors (*E. coli* DnaJ and GrpE, respectively). The Hsp70 system prevents protein aggregation and increases folding yields. Whether it also enhances the rate of folding remains unclear. Here we show that DnaK/DnaJ/GrpE accelerate the folding of the multi-domain protein firefly luciferase (FLuc) ~20-fold over the rate of spontaneous folding measured in the absence of aggregation. Analysis by single-pair FRET and hydrogen/deuterium exchange identified inter-domain misfolding as the cause of slow folding. DnaK binding expands the misfolded region and thereby resolves the kinetically-trapped intermediates, with folding occurring upon GrpE-mediated release. In each round of release DnaK commits a fraction of FLuc to fast folding, circumventing misfolding. We suggest that by resolving misfolding and accelerating productive folding, the bacterial Hsp70 system can maintain proteins in their native states under otherwise denaturing stress conditions.

## Abstract (web summary)

The Hsp70 system prevents protein aggregation and increases folding yields, but it is unknown whether it also enhances the rate of folding. Here the authors combine refolding assays, FRET and hydrogen/deuterium exchange-mass spectrometry measurements to study the folding of firefly luciferase and find that the bacterial Hsp70 actively promotes the folding of this multi-domain protein.

## Introduction

Hsp70 chaperones function as a central hub of the protein homeostasis network in bacteria and eukaryotic cells^1–3^. They recognize 5–7 amino acid sequence elements that are enriched in hydrophobic residues and are typically exposed by non-native protein substrates^1,2^. ATP-dependent binding and release of such segments allows Hsp70s to participate in a wide range of cellular processes, including protein folding, refolding, disaggregation, and protein transfer to cellular compartments or the proteolytic machinery^1^. Hsp70 proteins consist of an N-terminal nucleotide-binding domain (NBD) of ~40 kDa and a C-terminal substrate-binding domain (SBD) of ~30 kDa, connected by a hydrophobic linker. The SBD is composed of a β-sandwich domain, harboring the peptide-binding site, and an α-helical lid segment. Peptide substrate binds in an extended conformation in a groove in the β-sandwich domain^1,2,4^.

**Fig. 1:**
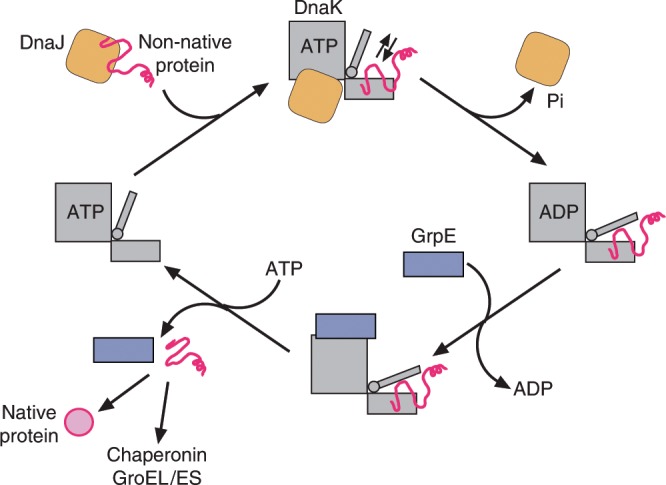
Dnak/DnaJ/GrpE reaction cycle. DnaJ captures the substrate protein and transfers it to DnaK in the ATP-bound state. DnaJ and substrate synergistically trigger ATP hydrolysis by DnaK, thereby generating a stable complex between the substrate and DnaK in the ADP-bound state. Catalysis of ADP-ATP exchange by GrpE stimulates client release and regenerates DnaK-ATP for another round of client engagement. Figure modified from ref. ^68^.

The activity of Hsp70 in protein folding is regulated by Hsp40 (or J-domain) proteins and nucleotide exchange factors, which coordinate a highly allosteric reaction cycle. This reaction cycle is best understood for the *E. coli* system, consisting of DnaK (Hsp70), DnaJ (Hsp40) and the nucleotide exchange factor GrpE (herein referred to as KJE) (Fig. 1). DnaJ is a chaperone that functions in recognizing and transferring substrate proteins to DnaK in the ATP state^5,6^, in which the hydrophobic inter-domain linker and the α-helical lid of the SBD are associated with the NBD, and the SBD is in an open conformation^7–10^. In this state DnaK has high on- and off-rates for substrate. Interaction of DnaJ with DnaK strongly accelerates (by >1000-fold) the hydrolysis of the bound ATP, generating the ADP state, in which SBD and NBD are loosely associated and the α-helical lid is closed, trapping the bound substrate (low on- and off-rates)^11,12^ (Fig. 1). Subsequent binding of GrpE to the NBD facilitates ADP-ATP exchange, opening the SBD and allowing substrate release for folding or transfer to downstream chaperones such as the chaperonin GroEL^6^. Rebinding to DnaK prevents off-pathway aggregation, with successive cycles resulting in high folding yields for proteins that would aggregate in the absence of chaperones^5,13,14^.

The KJE system mediates the folding of newly synthesized proteins and the refolding of proteins that unfolded under stress conditions such as heat stress, where DnaK and its co-chaperones are strongly induced^15–18^. Among the ~700 identified *E. coli* substrates of DnaK are numerous multi-domain proteins, as well as proteins that need to be stabilized for subsequent interaction with GroEL^17,19^. In de novo protein folding DnaK cooperates with the ribosome-binding chaperone Trigger factor, which acts upstream of DnaK^15,16,20^.

How exactly protein binding and release by Hsp70 translates into productive folding is not yet understood. Specifically, it remains to be determined whether and how this chaperone system modulates the energy landscape of folding reactions beyond preventing off-pathway aggregation. Indeed, it has been proposed that KJE can use the energy of ATP to catalytically unfold misfolded states^14,21,22^, and stabilize native states out of equilibrium under denaturing conditions^18,23^. A key question in this context is whether KJE accelerates protein folding under conditions in which neither the folding rate nor the yield is limited by aggregation. Here we addressed this question using firefly luciferase (FLuc) as a multi-domain model protein. FLuc is thermally unstable and highly aggregation-prone during folding, and due to its sensitive luminescence assay has been a preferred Hsp70 substrate in studies in vitro and in vivo^5,13,24,25^. We used single-pair fluorescence resonance energy transfer (spFRET) to study the folding of FLuc in the absence of confounding effects due to protein aggregation. This approach revealed that the *E. coli* Hsp70 system substantially accelerates FLuc refolding, and enabled direct detection of both spontaneous misfolding and conformational rescue of misfolded states by the KJE chaperone machinery. Hydrogen/deuterium exchange coupled to mass spectrometry (H/DX-MS) served to localize FLuc misfolding to the subdomain interface of the large N-terminal domain.

## Results

### The *E. coli* Hsp70 system accelerates FLuc folding

**Fig. 2:**
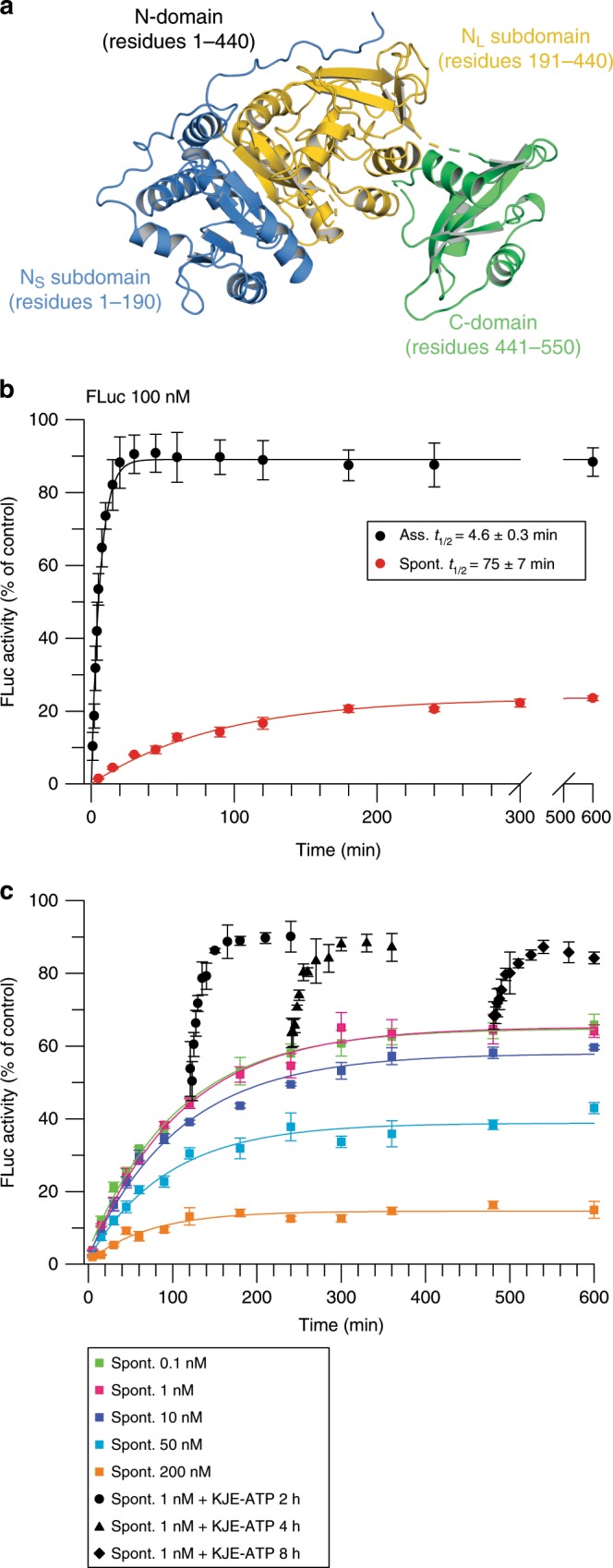
The KJE chaperone system accelerates FLuc folding. **a** Structure of FLuc (PDB ID: 1LCI) with domains indicated. **b** Spontaneous and chaperone-assisted folding of 100 nM FLuc was assayed upon dilution from 5 M GuHCl into buffer without or with KJE-ATP, respectively, by monitoring luminescence activity. KJE-mediated folding was performed with 3 µM DnaK, 1 µM DnaJ, 1.5 µM GrpE and 5 mM ATP. Error bars represent s.d. (*n* = 10). **c** Concentration dependence of FLuc refolding. FLuc was diluted from denaturant to different final concentrations (0.1–200 nM) and refolding assayed as in (**b**). Rescue of spontaneous folding reactions was assayed by adding KJE-ATP (0.3 µM DnaK, 0.1 µM DnaJ, 0.5 µM GrpE and 5 mM ATP) after 2, 4 or 8 h. Error bars represent s.d. (*n* = 3). Ass., KJE-ATP-assisted; Spont., spontaneous folding. Source data are provided as a Source Data file.

FLuc is a ~60 kDa protein consisting of a large N-domain (residues 1-440) and a smaller C-domain (residues 441-550)^26^ (Fig. 2a). The N-domain can be divided into small (N_S_; residues 1-190) and large (N_L_; residues 191-440) subdomains, of which the former has been shown to fold co-translationally^24,27^. DnaK/DnaJ/GrpE (KJE), as well as the eukaryotic Hsp70 chaperone system, can refold both heat- and chemically denatured FLuc^5,13,28,29^. To understand the mechanism of Hsp70-mediated folding, we monitored the rates of spontaneous and KJE-assisted folding of FLuc by sensitive luminescence assay^30^. Consistent with previous reports^31,32^, spontaneous renaturation was slow (*t*_1/2_ ~75 min), with aggregation limiting the folding yield to ~25% even at a concentration of 100 nM FLuc (Fig. 2b and Supplementary Table 1). In contrast, KJE-assisted folding was ~90% efficient and ~18-fold faster (*t*_1/2_ ~ 4 min).

To exclude aggregation as the cause of slow folding, we established the critical concentration for FLuc aggregation. Dual-color fluorescence cross-correlation spectroscopy (dcFCCS) showed absence of aggregation during refolding for equimolar mixtures of the labelled proteins at a total concentration below 10 nM (Supplementary Fig. 1a). Double-labelled FLuc (5 nM) served as positive control. Reducing the concentration of FLuc during refolding increased the yield of spontaneous folding up to ~60% (Fig. 2c and Supplementary Table 1). The folding rate remained unchanged, indicating that aggregation was not the cause but rather the consequence of slow folding. Note that the non-ionic detergent Tween 20 (T20; 0.05%) was used to prevent FLuc adsorption to tube walls during folding (Supplementary Fig. 1b, c). Refolding yield and kinetics were independent of the presence or absence of ATP (Supplementary Fig. 1d). The limited yield of renaturation indicated that a fraction of FLuc populates kinetically trapped folding intermediate(s) that do not convert to the native state within the time frame of our experiments (10 h).

To test whether kinetically trapped intermediates of FLuc (in the absence of aggregation at 1 nM FLuc) remained competent for refolding by KJE, we transferred aliquots of the reaction after different times of spontaneous folding (up to 8 h) into fresh tubes containing KJE-ATP. This resulted in accelerated folding to full yield (Fig. 2c). Without ATP, refolding in the presence of KJE occurred with a rate similar to that of the spontaneous reaction, but reached a yield of only ~40% (Supplementary Fig. 1e), apparently due to the binding of FLuc folding intermediate to DnaJ/DnaK. Addition of ATP resulted in accelerated folding to full yield (Supplementary Fig. 1e), consistent with the proposal that KJE can use the energy of ATP hydrolysis to shift the folding reaction out of equilibrium towards the native state^23^.

Together these findings demonstrate that the Hsp70 chaperone system actively promotes client protein folding in an ATP-dependent manner, by reversing and/or averting misfolding.

### Slow folding of FLuc is caused by inter-domain misfolding

**Fig. 3:**
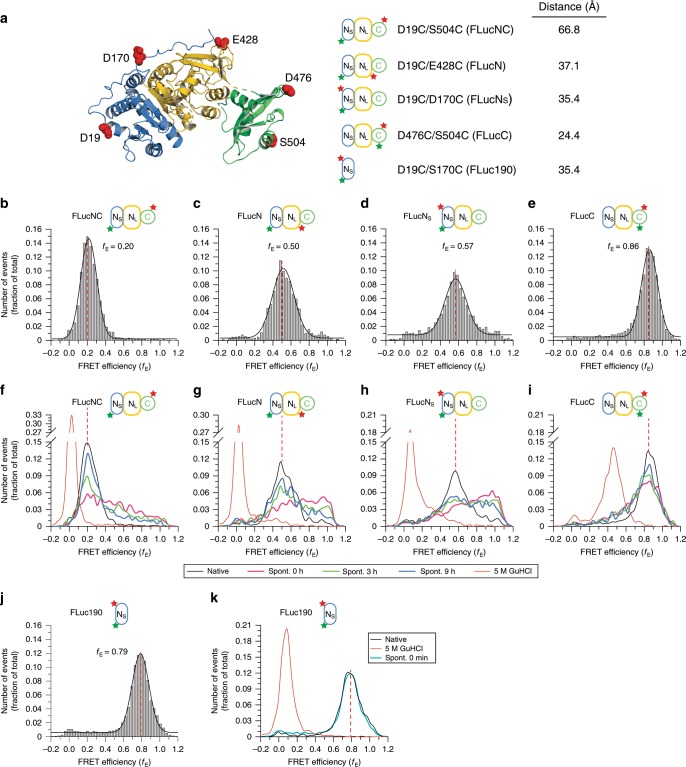
Slow spontaneous folding of FLuc is caused by inter-domain misfolding. **a** Structure of FLuc (PDB ID: 1LCI) showing fluorophore labelling positions. Mutations introduced to allow site-specific cysteine labelling are indicated, and estimated distances between dye pairs are given. FLuc variants were labelled with Atto532 (donor) and Atto647N (acceptor). **b**–**e** spFRET efficiency (f_E_) histograms of the native proteins: FLucNC (**b**), FLucN (**c**), FLucN_S_ (**d**) and FLucC (**e**). **f**–**i**, *f*_E_ histograms measured during spontaneous refolding of 50 pM denatured FLuc. spFRET was recorded for 1 h, either immediately (0 h) or at the indicated folding time points. **j**, **k**, spFRET efficiency (*f*_E_) histogram of the N_S_-subdomain (FLuc190) (**j**) and spontaneous folding of 50 pM denatured FLuc190 (**k**). Data were recorded for 30 min. Reactions b–k contained 5 mM ATP and 50 µM phenobenzothiazine (PBT). The *f*_E_ corresponding to the native state for each construct is indicated by a dashed vertical red line. Representative measurements of three independent repeats are shown. Spont., spontaneous folding. Source data are provided as a Source Data file.

To understand the basis of the slow and inefficient spontaneous folding of FLuc, we next monitored folding by spFRET combined with pulsed interleaved excitation (PIE)^33^. The spFRET experiments were performed at 50 pM FLuc, at which concentration FLuc is monomeric during refolding as measured by dcFCCS (Supplementary Fig. 1a). We designed four double-cysteine mutants of FLuc and labelled them with Atto532 and Atto647N as fluorescence donor and acceptor, respectively (*R*_o_ ~ 46 Å)^34^. Note that fluorophore attachment preserved FLuc foldability and activity (Supplementary Fig. 2a–d). FLucNC (D19C/S504C) reports on overall FLuc folding, FLucN (D19C/E428C) on folding of the N-domain, FLucN_S_ (D19C/S170C) on the N_S_-subdomain, and FLucC (D476C/S504C) on folding of the C-domain (Fig. 3a). We observed symmetric distributions of FRET efficiencies (*f*_E_) for each construct, with mean *f*_E_ values in good agreement with fluorophore distances predicted from the crystal structure (Fig. 3b–e). Upon dilution from denaturant (0 h time point), the FLucNC, FLucN and FLucN_S_ proteins converted from the low *f*_E_ state of the denatured protein to a broad *f*_E_ distribution (Fig. 3f–h). These distributions include conformations that are substantially more compact than the native state and thus are likely to contain non-native interactions (denoted as misfolded; MF), consistent with the detection of misfolded states of FLuc in optical tweezer experiments^35^. During folding these intermediates shifted towards the *f*_E_ distribution of the native state (Fig. 3f–h) with an apparent *t*_1/2_ of ~70–85 min (Supplementary Fig. 2e–g). In contrast, FLucC adopted the native state *f*_E_ at least 10-fold faster without populating detectable intermediates (Fig. 3e, i and Supplementary Fig. 2h), suggesting that the C-domain folds independently of the N-domain. Interestingly, rapid folding was also observed for the isolated N_S_-subdomain (FLuc190) (Fig. 3j, k), consistent with its ability to fold co-translationally^27,36^. This is in contrast to the slow folding of N_S_ in the context of full-length FLuc (FLucN_S_), which limits the folding yield (Fig. 3h, k and Supplementary Fig. 2g). Thus, folding of the N-domain is the rate-limiting step during spontaneous folding and involves misfolding between N_S_ and N_L_.

### KJE-assisted folding involves unfolding of misfolded states

**Fig. 4:**
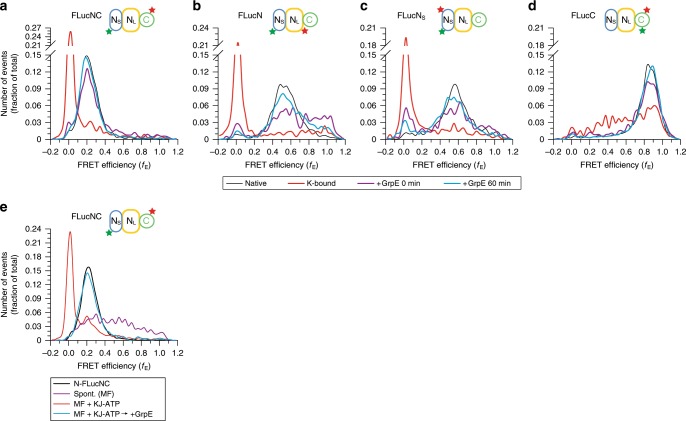
KJE reverses misfolding and commits bound protein to fast folding. **a**–**d** spFRET efficiency histograms measured during KJE-ATP assisted folding of FLucNC (**a**), FLucN (**b**), FLucN_S_ (**c**) or FLucC (**d**). GuHCl-denatured FLuc was diluted to 50 pM in buffer containing 0.3 µM DnaK, 0.1 µM DnaJ, 5 mM ATP and 50 µM PBT, and the *f*_E_ distribution of DnaK-bound FLuc was recorded, followed by initiation of folding with 0.5 µM GrpE. spFRET was recorded for 15 min either immediately (0 min) or after 60 min of folding. Representative measurements of three independent repeats are shown. **e** KJ-ATP converts compact, misfolded (MF) intermediates of FLuc to the DnaK-bound, expanded state. GuHCl-denatured FLucNC was diluted to 50 pM in buffer containing ATP and PBT, and the distance between N- and C-domains probed by spFRET as in (**a**). KJ-ATP were added after 1 h to generate DnaK-bound FLuc, followed by GrpE to initiate folding. Representative measurements of three independent repeats are shown. Spont., spontaneous folding. Source data are provided as a Source Data file.

How does the KJE chaperone system accelerate FLuc folding? To characterize the state of FLuc from which chaperone-assisted folding initiates, we diluted denatured FLuc into folding buffer containing DnaK, DnaJ and ATP (KJ-ATP). GrpE was omitted to prevent substrate release^5,6^. DnaJ allows for efficient transfer of protein substrate to DnaK and dissociates upon ATP hydrolysis, resulting in a stable DnaK-substrate complex with DnaK in the ADP state^2^ (Fig. 1). DnaK-bound FLucNC, FLucN and FLucN_S_ exhibited very low *f*_E_ distributions similar to those of the denatured proteins, indicative of highly expanded conformations (Fig. 4a–c). As expected, this effect required the function of DnaJ (Supplementary Fig. 3a). In contrast, the chaperones failed to stabilize FLucC in the expanded state (Fig. 4d), consistent with its rapid folding (Fig. 3i and Supplementary Fig. 2h). Importantly, the isolated N_S_-subdomain (FLuc190) also failed to interact with KJ-ATP (Supplementary Fig. 3b). Thus misfolding of N_S_ in the context of the complete N-domain results in exposure of DnaK binding sites, thereby allowing conformational expansion of the N_S_ subdomain by DnaK.

Analytical ultracentrifugation determined a mass of ~900 kDa for the DnaK-FLuc complex (Supplementary Fig. 4a), consistent with a diffusion coefficient of 21 µm^2^ s^−1^ by FCS (Supplementary Fig. 4b). dcFCCS measurements using an equimolar mixture of FLuc-Alexa532 and FLuc-Alexa532 demonstrated that the DnaK-FLuc complex contains only one molecule of FLuc (Supplementary Fig. 4c). Moreover, dcFCCS with FLuc-Alexa532 and DnaJ-Alexa647 showed no significant cross-correlation, indicating that the DnaK-FLuc complex contains essentially no DnaJ (<5% of complexes) (Supplementary Fig. 4c). The mass of ~900 kDa for the complex would correspond to ~12 DnaK bound per expanded FLuc molecule. This is consistent with the number of predicted DnaK binding motifs in FLuc (13 sites, Supplementary Fig. 4d, e)^37^. However, as DnaK can bind the flexible linker between the ATPase and peptide-binding domains of another DnaK^38^, not all DnaK molecules may be bound directly to FLuc. Folding of FLuc by the KJE system thus initiates from an unfolded state that resembles the denatured protein in terms of conformational expansion.

Addition of GrpE to the DnaK-bound FLuc resulted in a shift to the native spFRET distribution of FLuc (Fig. 4a–d). A minor population of expanded FLuc is apparent for FLucNC, FLucN and FLucN_S_ at early time points of the reaction, suggesting that non-native FLuc transiently re-engages the chaperone system during folding (Fig. 4a–c). Note that KJ-ATP do not bind native FLuc (N-FLuc) (Supplementary Fig. 5a). During assisted folding, FLuc also transiently populated compact intermediates similar to the MF states observed during spontaneous folding (see Fig. 4b). To understand the fate of these intermediates during KJE cycling, we first generated compact folding intermediates of FLucNC in the absence of chaperones, and then added KJ-ATP (Fig. 4e). The broad *f*_E_ of the folding intermediates shifted to the narrow, low *f*_E_ characteristic of the DnaK-bound state (Fig. 4e). Note that the small amount of spontaneously folded FLucNC did not shift (Fig. 4e), as KJ-ATP does not bind N-FLuc (Supplementary Fig. 5a). Subsequent addition of GrpE resulted in efficient folding from the expanded state (Fig. 4e). A broad conformational distribution of folding intermediates (including MF states) also formed during spontaneous folding of FLuc at 37  ^o^C, followed by expansion upon chaperone-binding (Supplementary Fig. 5b). Together these data show that the mechanism of KJE-assisted folding involves unfolding of kinetically trapped misfolded states.

### FLuc misfolding involves the interface of N_S_ and N_L_ subdomains

**Fig. 5:**
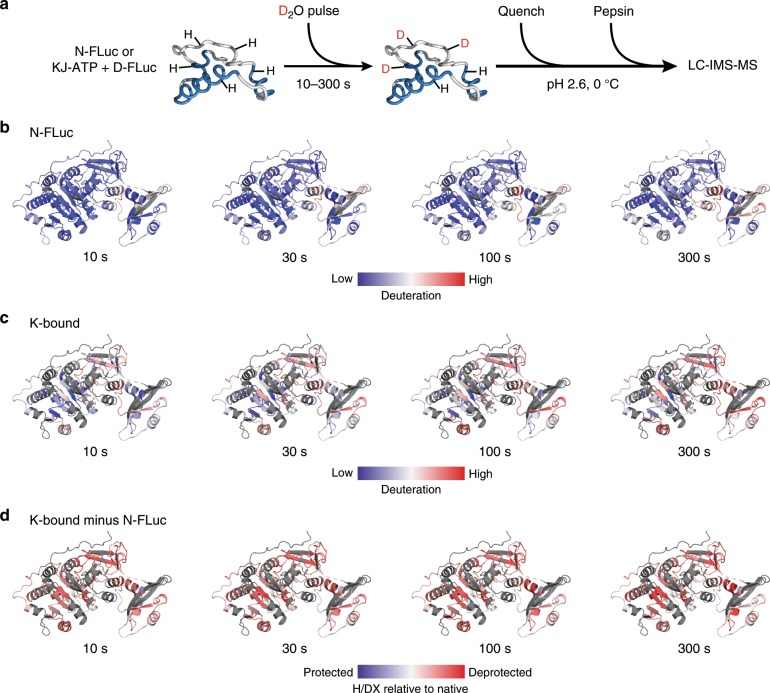
Conformational dynamics of native and DnaK-bound FLuc. **a** Scheme of equilibrium H/DX-MS experiment. **b**–**c**, Peptide-level deuterium exchange of native FLuc (N-FLuc) (**b**) or DnaK-bound FLuc (K-bound) (**c**) after exposure to deuterium for 10-300 s. Relative fractional deuterium exchange for each peptide is mapped onto the structure of FLuc (PDB ID: 1LCI) as a gradient from blue (0%) to red (75%). **d** Difference in deuterium exchange between DnaK-bound and N-FLuc at peptide level. Deuteration differences are scaled from blue (−50%) to red (+50%). Regions coloured red are deprotected when bound to DnaK. H/DX data are the average of three independent repeats.

To extend our insight into FLuc folding from domain to peptide level, we analysed KJE-assisted folding reactions using H/DX-MS^39,40^. Note that H/DX-MS of spontaneous folding was not possible, because FLuc aggregates at the higher concentrations (1 μM) required for these experiments. KJE-ATP-dependent refolding (10 μM DnaK/3.3 µM DnaJ/5 µM GrpE) occurred with a yield of ~76% and a rate comparable to that measured at lower FLuc concentration (Supplementary Fig. 6). We first compared the conformational properties of DnaK-bound and N-FLuc by D_2_O pulse analysis at equilibrium (Fig. 5a). Peptide coverage was ~95% for N-FLuc and ~63% for the DnaK-bound protein (Supplementary Fig. 7a, b and Supplementary Data 1a). DnaK-bound FLuc was globally destabilized relative to the native protein (Fig. 5b–d and Supplementary Data 1b). The majority of peptides exchanged in the EX2 regime, and several peptides showed evidence of EX1 or mixed EX1/EX2 exchange kinetics in the DnaK-bound state (Supplementary Fig. 8a–c). Correlated exchange at the EX1 limit is evidence of cooperative local unfolding^40^. The observation that parts of DnaK-bound FLuc sample both unfolded and partially folded states is consistent with the detection by nuclear magnetic resonance (NMR) of transient secondary structure in a small protein domain bound to DnaK^41^.

**Fig. 6:**
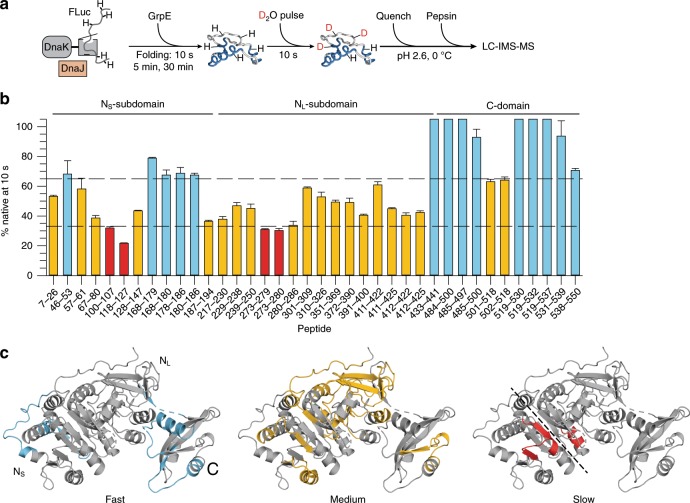
KJE-assisted folding of FLuc at peptide resolution. **a** Scheme of H/DX-MS experiment. DnaK is shown schematically in grey and DnaJ in orange. **b** Fraction of native deuterium exchange reached by FLuc peptides after 10 s folding. D uptake at 10 s was normalized to the difference in D uptake between K-bound and N-FLuc for each peptide. Only peptides exhibiting a >1 Da difference between the DnaK-bound and native states are shown. Peptides are characterized as fast (upper third, blue), medium (middle third, gold) or slow (lower third, red) folders, based on their relative protection compared to N-FLuc. Error bars represent s.d. (*n* = 3). Source data are provided as a Source Data file. **c** Location of the peptides colour-coded as in (**b**), indicated on the structure of FLuc. Dotted line indicates the N_S_–N_L_ domain interface.

We next probed the conformation of FLuc by 10 s D_2_O pulses at 10 s, 5 min and 30 min after initiating folding with GrpE (Fig. 6a). Consistent with the folding kinetics measured by luminescence assay, all peptides analysed (Supplementary Fig. 7c) acquired native protection levels within 30 min (Supplementary Data 1c). Bimodal peptide-mass distributions indicative of co-existing native and non-native populations were observed at intermediate folding times (Supplementary Fig. 9). Analysis of H/DX protection after 10 s revealed that the C-domain and part of the N_S_-subdomain (residues 46-53 and 168-186) acquire native-like protection rapidly, followed by the remainder of the N-domain (Fig. 6b, c). Strikingly, the peptides that acquire protection at the slowest rate (residues 100-107, 118-127 and 273-280) are located at the interface between the N_S_- and N_L_-subdomains (Fig. 6b, c). Formation of this interface is therefore rate limiting for overall folding, with misfolding apparently resulting in compact states of FLuc that must be resolved by a further round of chaperone action (Fig. 4e). Indeed, predicted DnaK binding motifs on FLuc are enriched at the N_S_–N_L_ interface (5 of 13 high confidence sites, Supplementary Fig. 4d, e), suggesting that non-native conformations that expose this region are efficiently targeted by DnaK.

### DnaK commits a fraction of bound protein to fast folding

**Fig. 7:**
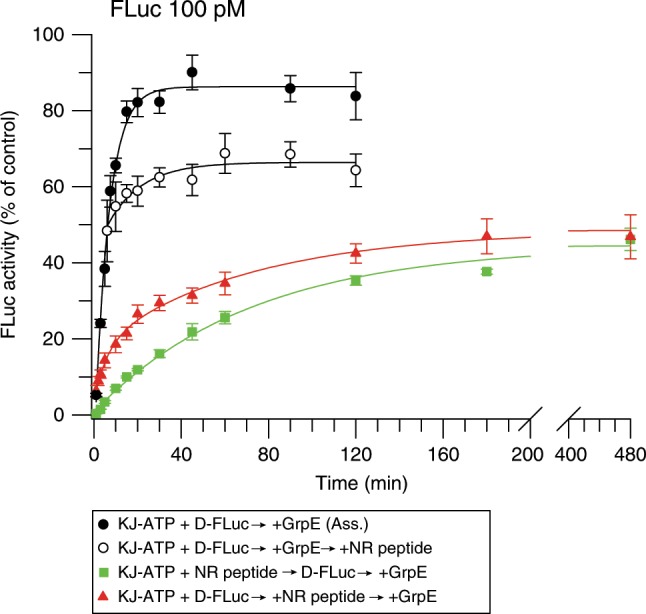
DnaK commits a fraction of bound FLuc to fast folding. Folding of FLuc in a single-round of chaperone action. Rebinding of FLuc to DnaK was inhibited using excess NR peptide (125 µM). For single round folding, GuHCl-denatured FLuc was diluted to 100 pM in buffer containing KJ-ATP, followed by addition of NR and initiation of folding with GrpE (red triangles). As controls, NR was either omitted (black circles) or added to KJ-ATP before FLuc (green squares), or after 5 min of chaperone-assisted folding (white circles). Error bars represent s.d. (*n* = 3). Ass., KJE-ATP-assisted. Source data are provided as a Source Data file.

To understand the mechanism of accelerated folding by the KJE system, it was of interest to measure the yield and rate of folding for a single round of DnaK binding and release. We addressed this question by inhibiting the rebinding of FLuc to DnaK after GrpE-mediated release. This was achieved by addition of the peptide GSGNRLLLTG (NR) that competes for the substrate-binding site on DnaK^4,42,43^. When excess NR was added to KJ-ATP before denatured FLuc, folding occurred at the slow rate of the spontaneous reaction (Fig. 7), indicating inhibition of DnaK binding. Note that the reduced yield of folding (~40%) is due to the presence of DnaJ, which is not efficiently blocked by NR (see also Supplementary Fig. 1e). Addition of NR after 5 min of KJE-assisted folding caused an immediate shift of the accelerated folding reaction to the slow kinetics of spontaneous folding (Fig. 7). To assess the folding reaction associated with a single chaperone cycle, we added NR to a pre-formed DnaK-FLuc complex, and then initiated chaperone release with GrpE. This resulted in biphasic folding kinetics, with a fast phase (*t*_1/2_ = 4 ± 1 min) identical to the folding rate in the cycling KJE reaction, and a slow phase (*t*_1/2_ = 49 ± 8 min) close to the rate of spontaneous folding (Fig. 7). Approximately 15% of FLuc reached native state during the fast phase, compared to only ~3% in the equivalent time of spontaneous refolding (Fig. 7). Thus, the Hsp70 mechanism provides access to a fast folding trajectory for a fraction of FLuc molecules in each chaperone cycle, perhaps favoring a mechanism in which segments of the N_S_-subdomain are released for fast folding (Fig. 6b), while N_S_–N_L_ interface regions remain DnaK-bound. These molecules apparently by-pass the non-native interactions within the N-domain that are rate-limiting for spontaneous folding and are committed to fold rapidly to the native state.

### KJE shifts the folding equilibrium towards the native state

**Fig. 8:**
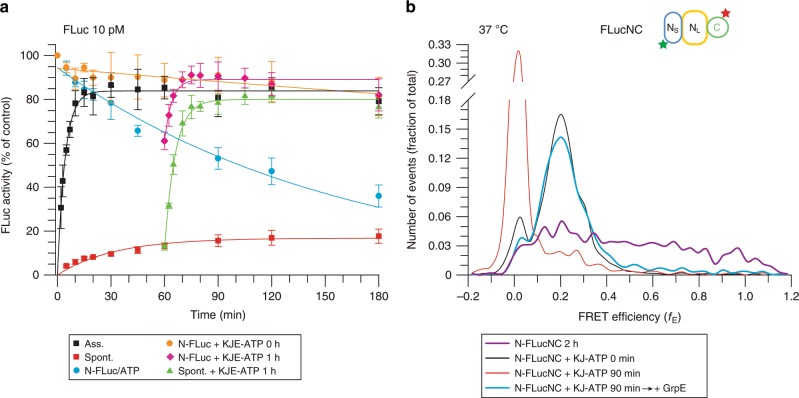
KJE-accelerated folding maintains the native state of FLuc. **a** Luminescence activity of 10 pM FLuc was monitored at 37 ^o^C, in the presence or absence of KJE-ATP. Time-dependent loss of FLuc activity was monitored by shifting N-FLuc from 25 ^o^C to 37 ^o^C (blue circles). 1 µM DnaK, 0.33 µM DnaJ and 1.5 µM GrpE were included during temperature upshift of N-FLuc (orange circles), or added after 1 h incubation of N-FLuc at 37 ^o^C (magenta diamonds), or after 1 h of spontaneous refolding from denaturant at 37^o^C (green triangles). Assisted (Ass., black squares) and spontaneous (Spont., red squares) refolding are also shown. Error bars represent s.d. (n = 5). Ass., KJE-ATP-assisted; Spont., spontaneous folding. **b** N-FLucNC (10 pM) in folding buffer with PBT and ATP was shifted from 25 ^o^C to 37 ^o^C in the presence or absence of KJ. spFRET was recorded for 30 min after 2 h without chaperones. Upon heating in the presence of KJ-ATP, spFRET was recorded either immediately (0 min) or after 90 min. GrpE was added after 2 h to initiate refolding of FLuc from the DnaK-bound state. Representative measurements of three independent repeats are shown. Source data are provided as a Source Data file.

To test whether accelerated folding by KJE-ATP may be important in stabilizing proteins under conditions of heat stress, we took advantage of the limited thermal stability of FLuc^13,44^. Incubation of N-FLuc at 37 ^o^C resulted in loss of activity over time (Fig. 8a). Note that aggregation was avoided by using a very low FLuc concentration (10 pM) (Supplementary Fig. 10). Spontaneous folding at 37 ^o^C was slow and inefficient, with a final yield of only ~15% (Fig. 8a). In contrast, KJE-assisted folding reached ~85% (*t*_1/2_ = 2.9 ± 0.3 min). Addition of KJE-ATP to the spontaneous folding reaction or thermally denatured FLuc rapidly restored full activity (Fig. 8a), similar to the observations at 25 ^o^C (Fig. 2c and Supplementary Fig. 1e). When N- FLuc was incubated at 37 ^o^C together with KJE-ATP, activity was maintained (Fig. 8a). Upon thermal unfolding in the absence of chaperones, FLucNC populated a broad conformational distribution of folding intermediates by spFRET (Fig. 8b). In contrast, in the presence of KJ-ATP, the f_E_ distribution of native FLucNC (N-FLucNC) gradually shifted to that of the expanded DnaK-bound state, from which it refolded upon addition of GrpE (Fig. 8b).

These results demonstrate the ability of the KJE chaperone system to use the energy of ATP to shift the folding equilibrium of proteins like FLuc towards the native state under denaturing conditions, consistent with recent observations^18,23^. Our findings indicate that KJE performs this function by unfolding kinetically trapped, misfolded states and allowing re-folding to the native state along a fast trajectory that circumvents misfolding.

## Discussion

**Fig. 9:**
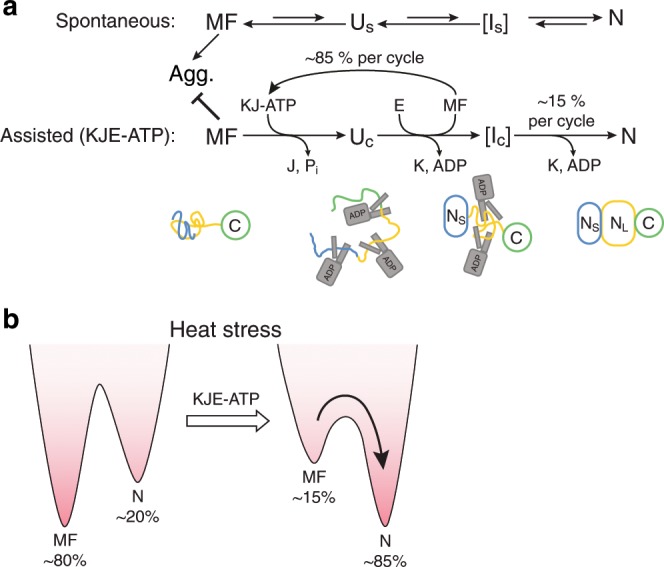
Model of spontaneous and KJE-ATP assisted folding of FLuc. **a** MF, misfolded compact intermediate; Agg., aggregated protein; U_S_, unfolded state in the absence of chaperones; I_S_, folding intermediate of spontaneous folding; U_C_, unfolded state in complex with DnaK; I_C_, intermediate committed to fast folding; N, native state. Lengths of arrows reflects approximate relative rates. **b** Simplified energy diagrams to illustrate the effect of KJE-ATP in stabilizing the native state of FLuc at elevated temperature by accelerating refolding of MF.

In the canonical model of Hsp70 function, nucleotide-driven cycles of binding and release inhibit aggregation of the client protein without influencing its folding pathway. Here we demonstrated an additional function of Hsp70 in accelerating the folding of a model multi-domain protein and analysed its mechanism. During spontaneous folding in the absence of aggregation, FLuc populates kinetically trapped, misfolded states (MF). These intermediates are aggregation-prone but remain monomeric at very low protein concentration. They are characterized by non-native interactions between the N_S_ and N_L_ subdomains of FLuc, which need to be resolved to allow N-domain folding (Fig. 9a). KJE-ATP markedly (~20-fold) accelerates folding by two complementary activities. First, DnaK and DnaJ cooperate in an ATP hydrolysis-dependent process to unfold misfolded FLuc, thereby catalyzing escape from kinetic traps. Binding of multiple DnaK molecules converts MF to an expanded state (U_C_) (Fig. 9a). Similar expanded conformations for other Hsp70-bound proteins have been suggested to arise from steric repulsion between multiple DnaK molecules bound to the same substrate protein^41,45–47^. Second, GrpE-mediated release of DnaK directs the bound FLuc molecules towards an intermediate conformation, I_C_, that is committed to fold fast to the native state (Fig. 9a), effectively lowering the energy barrier towards N (Fig. 9b). As shown by the single cycle experiments, this reaction is critical for acceleration of FLuc folding but is of limited efficiency (~15% of molecules). The remainder of FLuc reverts to MF states after release from DnaK, followed by unfolding upon rebinding to DnaK.

At an efficiency of ~15% for fast folding per chaperone cycle, ~4 cycles are required for half-maximal folding. Each cycle would nominally consume ~10 molecules of ATP, assuming that ~10 DnaK bind to FLuc. However, FLuc molecules may not necessarily return to the fully expanded, DnaK-bound state during uninterrupted cycling and fewer DnaK may be sufficient to destabilize MF. The actual ATP consumption of the folding reaction remains to be determined.

Prior work provided evidence that DnaK can use chemical energy from ATP hydrolysis to drive substrate folding out of equilibrium^23,48^. Our data are consistent with this concept, and demonstrate that in case of FLuc, stabilization of the native state can be explained by kinetic folding assistance, whereby KJE-ATP selectively accelerates the re-folding of kinetically trapped, misfolded protein (Fig. 9). In the absence of chaperone, these kinetically trapped states function effectively as a sink, especially under conditions that destabilize the native protein, such as elevated temperature.

Our observation that misfolding is avoided by a fraction of FLuc in each chaperone cycle would appear inconsistent with a mechanism of simultaneous release of the multiple bound DnaK molecules. Instead, and consistent with the H/DX-MS data, we hypothesize that KJE biases the folding pathway by a mechanism of asynchronous release of DnaK molecules. Additional support for this idea comes from NMR experiments on DnaK-bound hTRF1, a 53-residue model client^49,50^. hTRF1 can bind between one and three DnaK, which is suggested to result in conformational heterogeneity in the bound substrate at the onset of folding. A mechanism of asynchronous release might allow structure formation in the N_S_-subdomain of FLuc without interference from the N_L_-subdomain, thereby bypassing the kinetic folding trap for a fraction of molecules in each cycle (Fig. 9a). The type of inter-domain misfolding rescued by the KJE system is common in large multi-domain proteins^36,51–53^ and artificial poly-proteins^21,35,54,55^, and might occur during de novo folding or upon heat stress-induced denaturation. Indeed, the KJE system has recently been shown to stabilize numerous multi-domain proteins against thermal denaturation in vivo (Fig. 9b)^18^. Our observations for the heterologous substrate FLuc may therefore more generally explain the stress-protective function of Hsp70.

The ability to accelerate protein folding by an ATP-dependent mechanism has also been demonstrated for the cylindrical GroEL/ES chaperonin^56^. Several proteins utilizing this system have been shown to populate dynamic folding intermediates that convert only slowly to the native state due to a long search time for the formation of native contacts^57–59^. Their encapsulation in the folding cage formed by GroEL and GroES results in a rate enhancement of folding by one to two orders of magnitude^57,58,60,61^, which has been attributed to an effect of entropic confinement^56–58^. In contrast, KJE-mediated binding and release cycles fail to accelerate the folding of these proteins but rather slow their folding kinetics^19,58,60^. Emerging from these findings is the concept that KJE and GroEL/ES, as major chaperone systems in the bacterial cytosol, attend to protein subsets that populate different types of kinetically trapped folding intermediates. While GroEL/ES catalyses folding of entropically stable intermediates, Hsp70 rescues misfolded states that are stabilized by non-native interactions that need to be resolved in order to allow folding.

## Methods

### Cloning, plasmids and strains

Full-length FLuc from *Photinus pyralis* was cloned into a pET3a plasmid (Supplementary Table 2). Point mutations for site-specific fluorophore labelling were introduced using the QuikChange Multi Site-Directed Mutagenesis Kit (Agilent Technologies). FLuc190 was generated by insertion of a stop codon using Q5 Site-Directed Mutagenesis Kit (New England BioLabs). GrpE was expressed from plasmid pET3a, and DnaK and DnaJ from pET11d. Plasmids, proteins and strains used or generated in this study are listed in Supplementary Table 3.

### Expression and purification of FLuc and variants

Wild-type FLuc was purchased from Promega, or purified as follows. Wild-type and mutant FLuc constructs were transformed into *E. coli* BL21 (DE3) cells. Cells were grown at 37 °C until OD_600_ reached ~0.4. The temperature was then shifted to 18 °C and cultures were incubated for 1 h before addition of IPTG to a final concentration of 0.25 mM. Proteins were expressed for 16 h at 18 °C. Cells were harvested by centrifugation at 5000*g* for 30 min and pellets were resuspended in lysis buffer (50 mM NaH_2_PO_4_ pH 8.0, 300 mM NaCl, 10 mM Imidazole, 1 mg mL^−1^ lysozyme, PMSF, aprotinin, PepA, leupeptin, DNase I and Complete^TM^, EDTA free protease inhibitor cocktail (Roche)). Cells were lysed by sonication, and the lysate was cleared by centrifugation at 66,000*g* for 30 min. The supernatant was loaded onto a 5 mL Ni-NTA column (HisTrap^TM^HP, GE Healthcare Life Sciences) pre-equilibrated with equilibration buffer (50 mM NaH_2_PO_4_ pH 8.0, 300 mM NaCl, 20 mM imidazole). Protein was eluted in 2 mL fractions using equilibration buffer containing 500 mM imidazole, and purity of the fractions was assessed by 12% SDS-PAGE. Fractions containing pure FLuc were pooled, concentrated, and buffer exchanged into FLuc storage buffer (25 mM Tris-acetate pH 7.8, 1 mM EDTA, 0.2 M ammonium sulfate, 15% glycerol, 30% ethylene glycol, 2 mM TCEP) before snap-freezing in liquid N_2_ and storage at −80 °C.

### Expression and purification of chaperones

The DnaK construct was transformed into *E. coli* BL21 (DE3) cells. Cells were grown at 37 °C until OD_600_ reached 0.6–0.8, then expression was induced with 0.5 mM IPTG at 30 °C. After 4–5 h expression, cells were harvested, resuspended in buffer (20 mM Tris-HCl pH 7.4, 1 mM EDTA, 1 mg mL^−1^ lysozyme, PMSF, aprotinin, PepA, leupeptin, DNase I and Complete^TM^, EDTA free protease inhibitor cocktail (Roche)) and lysed as described above for FLuc by centrifugation at 5000*g* for 30 min. Cells were resuspended in buffer A (20 mM Tris-HCl, pH 7.4, 1 mM EDTA). The lysate was clarified as described above, and the supernatant was loaded onto a Source 30Q (GE Healthcare, 50 mL) column pre-equilibrated with buffer A. The column was washed with buffer A, and protein was eluted using buffer A/1 M NaCl. Fractions containing DnaK were pooled and buffer exchanged into HMK buffer (20 mM Hepes/KOH, pH 7.4, 5 mM Mg(OAc_2_)) using a HiPrep 26/10 desalting column (GE Healthcare, 56 mL) column. The sample was then loaded onto a Hiprep Heparin Sepharose FastFlow FF16/10 column (GE Healthcare) pre-equilibrated with HMK buffer. Elution was performed with a 0–500 mM NaCl gradient. Fractions containing DnaK were pooled and buffer exchanged into HMK buffer, then loaded onto a MonoQ 10/100 column (GE Healthcare). Protein was eluted with a 0–500 mM NaCl gradient, and pooled fractions were buffer exchanged into HMK buffer/100 mM NaCl. The eluted protein was further purified by gel filtration using a Superdex 200 16/60 column equilibrated with HMK buffer/100 mM NaCl. Fractions containing pure DnaK were pooled and concentrated before snap-freezing in liquid N_2_ and storage at −80 °C.

The GrpE construct was transformed into *E. coli* BL21 (DE3) cells. Expression was performed as described above for DnaK. Harvested cells were resuspended in buffer (50 mM Tris-HCl pH 8.0, 10% sucrose (w/v)) and lysed with lysozyme solution containing 46 µg mL^−1^ spermidine, 50 mM DTT, 50 mM EDTA and 0.2 g mL^−1^ ammonium sulfate. For lysis, the cell suspension was incubated for 45 min on ice, then heated to 37 °C for 5 min with gentle shaking, followed by cooling on ice for 5 min. The lysate was clarified by centrifugation at 66,000*g* for 30 min at 4 °C. Ammonium sulfate was then added to a final concentration of 0.35 g L^−1^ and the solution was stirred for 20 min. Following centrifugation (66,000*g*, 30 min), the pellet was resuspended in buffer B (25 mM Hepes-KOH pH 8.0, 1 mM EDTA, 10 mM 2-mercaptoethanol, 20% (v/v) glycerol) and loaded onto an Enrich Q column (BioRad) pre-equilibrated with buffer B. The protein was eluted with buffer C (25 mM Hepes-KOH pH 8, 1 mM EDTA, 10 mM 2-mercaptoethanol, 10% (v/v) glycerol, 2 M KCl) and then buffer exchanged into buffer B before performing a second round of ion exchange chromatography using a MonoQ column (GE Healthcare). The eluted protein was further purified by gel filtration using a HiPrep16/60 Sephacryl-S100 column (GE Healthcare) pre-equilibrated with buffer B. Fractions containing pure GrpE were pooled and concentrated before snap-freezing in liquid N_2_ and storage at −80 °C.

DnaJ was expressed and purified as described previously^6^.

### Luminescence assay of FLuc folding

Denatured FLuc was prepared by incubation of native FLuc (N-FLuc) in 5 M GuHCl and 10 mM DTT for 1 h at 25 °C. Spontaneous folding was initiated by 100-fold dilution into folding buffer (25 mM Hepes-KOH pH 7.5, 100 mM KCl, 10 mM Mg(OAc)_2_, 2 mM DTT, 0.05% T20), resulting in a final GuHCl concentration of 50 mM. Assisted folding reactions were initiated by 100-fold dilution into folding buffer containing DnaK, DnaJ, GrpE and 5 mM ATP. Chaperone concentrations were optimized to maximize refolding yield at the concentration of FLuc used and avoid inhibitory effects of excess chaperones^29^. Reactions with 100 nM FLuc contained 3 µM DnaK, 1 µM DnaJ and 1.5 µM GrpE. Reactions with 1 nM, 0.1 nM or 50 pM FLuc contained 0.3 µM DnaK, 0.1 µM DnaJ and 0.5 µM GrpE. Reactions with 10 pM FLuc (at 37 °C) contained 1 µM DnaK, 0.33 µM DnaJ and 1.5 µM GrpE. Reactions were performed at 25 °C unless otherwise indicated.

Luminescence was measured using a Lumat LB9508 luminometer (Berthold Technologies). At indicated time points, aliquots of folding reactions or N-FLuc were diluted at least 25-fold in Luciferase Assay System solution (Promega) and luminescence was measured for 2 s at 25 °C. Folding yields were calculated by normalizing to the luminescence activity of N-FLuc. Prior to analysis, N-FLuc stocks were centrifuged at 20,000*g* for 30 min to remove aggregates. All measurements were performed at least three times. Folding curves were fit using Sigmaplot 14.0 (Systat Software).

Rescue of FLuc activity was performed by adding 0.3 µM DnaK, 0.1 µM DnaJ, 0.5 µM GrpE and 5 mM ATP at 2 h, 4 h or 8 h after initiation of spontaneous folding as described above.

To analyse FLuc folding in a single round of chaperone action, GuHCl-denatured FLuc was diluted to 100 pM in folding buffer containing 0.3 µM DnaK, 0.1 µM DnaJ, 5 mM ATP and 125 μM peptide NR peptide (GSGNRLLLTG). After incubation for 1 min, luminescence activity was measured at intervals as described above. As controls, peptide NR was added to the chaperone mixture prior to addition of FLuc, or after 5 min of chaperone-assisted folding.

Reactions at 37 °C were performed as described above, except that folding was assayed at 10 pM FLuc, and assisted folding reactions contained 1 µM DnaK, 0.33 µM DnaJ and 1.5 µM GrpE.

### Fluorophore labelling of proteins

For spFRET experiments, double-cysteine mutant variants of FLuc were labelled with Atto532-maleimide and Atto647N-maleimide (ATTO-TEC) at a 1.5-fold molar excess of each fluorophore. Before labelling, FLuc cysteine residues were reduced by incubation with 2 mM tris(2-carboxyethyl)phosphine (TCEP), which was subsequently removed using a Nap5 column (GE Healthcare). Labelling was performed in labelling buffer (25 mM Hepes-KOH pH 7.5, 100 mM KCl, 10 mM Mg(OAc)_2_, 5 mM ATP, 100 µM phenylbenzothiazole (PBT)) for 2 h on ice. After labelling, free dye was removed using a Nap5 column pre-equilibrated with FLuc storage buffer. Labelling efficiencies were typically ~70%, and the absence of free dye was confirmed using FCS. The specificity of labelling was assessed by comparing the measured FRET efficiency distribution with the distance between labelling residues according to the crystal structure of FLuc (PDB: 1LCI)^26^.

For FCS and FCCS experiments, wild-type FLuc was labelled at the N-terminus with either Alexa532 or Alexa647 N-hydroxysuccinimide (NHS) ester (Molecular Probes) at a 5-fold molar excess. Labelling was performed at 4 °C for 90 min in 0.1 M NaHCO_3_ pH 8.3, followed by removal of free dye using a Nap5 column pre-equilibrated with FLuc storage buffer. Labelling efficiencies were typically ~75%. Double-labelled FLuc for FCCS experiments was prepared by incubation of FLucNC (D19C/S504C) with Alexa532- and Alexa647-maleimide. Labelling was performed as described above for the spFRET constructs.

For FCS experiments, FLucNC was labelled with Atto647N-maleimide as described above for spFRET constructs.

For analytical ultracentrifugation experiments, FLucNC was labelled with a 6-fold excess of Atto532-maleimide. Labelling was performed in labelling buffer containing 4 M GuHCl, and without PBT, for 1 h at 25 °C. Free dye was removed using a Nap5 column equilibrated with labelling buffer containing 4 M GuHCl.

DnaJ was labelled at the N-terminus with Alexa647 N-hydroxysuccinimide ester (Molecular Probes) at a 2-fold molar excess. Labelling was performed at 4 °C for 90 min in 0.1 M NaHCO_3_ pH 8.3, and free dye was removed using a Nap5 column pre-equilibrated with buffer (25 mM Hepes-KOH pH 7.5, 100 mM KCl, 10 mM Mg(OAc)_2_, 2 mM DTT, 10% (v/v) glycerol). Labelling efficiencies were typically ~80%.

### Fluorescence correlation and cross-correlation spectroscopy

FCS and dcFCCS measurements using pulsed interleaved excitation (PIE)^33^ were performed on a MicroTime 200 inverse time-resolved fluorescence microscope (PicoQuant) as previously described^62^. Experiments were performed at a constant temperature of 20 °C unless otherwise stated. Pulsed diode lasers at 530 nm (LDH-P-FA-530) and 640 nm (LDH-PC-640B) were used for excitation of Alexa532 and Alexa 647, respectively. For FCS measurements, the laser power was set to 60 µW measured before the major dichroic filter. For FCCS measurements, each laser was set to 40 µW. The lasers were pulsed with a rate of 26.7 mHz. Measurements were performed using a water immersion objective (×60 1.2 NA, Olympus) in the sample cuvette (Ibidi). The emitted fluorescence was separated from excitation light by a dichroic mirror (Z532/635RPC), guided through a pinhole (75 µm) and in case of cross-correlation split according to wavelength by a beam splitter (600 DCXR) onto photon avalanche diodes (SPADs) (PDM series, MPD). The emission light was filtered by emission bandpass filters (HQ 690/70 and HQ 580/70, Chromas) in front of the respective detector. Detection was performed using time correlated single photon counting, making it possible to correlate any given photon with the excitation source. For FCS measurements, after-pulsing artifacts were removed by fluorescence lifetime filters (SymPhoTime, PicoQuant)^63^. Correlation plots were fitted with triplet diffusion equation using SymPhoTime 32 (PicoQuant). The confocal volume (V_eff_) was calibrated daily using Atto655-maleimide dye.

The diffusion time of 50 pM FLucNC-Atto532 was measured by FCS for FLuc in the native state or in complex with DnaK, prepared as described above for the folding assays. Autocorrelation was recorded for 30 min.

FCCS measurements were performed to measure intermolecular association during spontaneous folding of FLuc. FLuc-Alexa532 and FLuc-Alexa647 were denatured as described above, then diluted to different final concentrations in folding buffer, maintaining a 1:1 molar ratio of labelled proteins. Where indicated, reactions were supplemented with different concentrations of denatured, un-labelled FLuc. FCCS was recorded for 30 min at 25 °C, or 1 h at 37 °C. Reactions were held at 25 °C or 37 °C, as indicated, using a temperature controlled chamber (Ibidi Heating System). Double-labelled FLucNC (5 nM) was used as a positive control.

To determine the composition of the chaperone-FLuc complex, complexes were prepared as described above, using pairs of labelled components, and FCCS was recorded for 30 min. Complexes with labelled DnaJ were prepared with 0.3 µM DnaK, 10 nM DnaJ-Alexa647, 90 nM un-labelled DnaJ and 10 nM FLuc-Alexa532. To test whether complexes contained multiple FLuc molecules, complexes were prepared with 0.3 µM DnaK, 0.1 µM DnaJ, 50 pM FLuc-Alexa532 and 50 pM FLuc-Alexa647. Double-labelled FLucNC-Alexa532/647 at 50 pM was used as a positive control.

### Single-pair fluorescence resonance energy transfer (spFRET)

spFRET experiments were performed on a MicroTime 200 inverse time-resolved fluorescence microscope (PicoQuant) using pulsed interleaved excitation as described above. Temperature was maintained at 20 °C unless otherwise stated. Each laser was set to 40 µW, measured before the major dichroic filter. Unless otherwise stated, all spFRET experiments were performed at 50 pM FLuc to ensure that the probability of having more than one particle in the confocal volume is < 1%. FRET was recorded for 15 min (chaperone-assisted folding at 20 ^o^C), 30 min (chaperone-assisted folding at 37 ^o^C) or 1 h (spontaneous folding). Assisted folding reactions contained 0.3 µM DnaK, 0.1 µM DnaJ and 0.5 µM GrpE where indicated. All experiments were performed in folding buffer containing 5 mM ATP and 50 µM PBT and performed at least three times (residual GuHCl 50 mM).

Data were analysed using a burst intensity approach^64^ in SymPhoTime (PicoQuant). A single molecule diffusing through the confocal observation volume results in a burst in fluorescence intensity. A burst was considered a significant event when it contained more than 25 photons in a 1 ms bin time window. In addition, a threshold of 15 photons following red excitation was used to confirm the presence of a functional acceptor fluorophore. To constitute a significant measurement, at least 500 events were analysed for a single spFRET distribution histogram. FRET efficiencies were calculated from fluorescence intensities of donor (*I*_D_) and acceptor (*I*_A_) fluorophore by the equation:

1 $${{E = }}\frac{{{{I}}_{\mathrm{A}}}}{{{{I}}_{\mathrm{A}} + \gamma {{I}}_{\mathrm{D}}}},$$

where γ = (Φ_A_η_A_/Φ_D_η_D_) denotes a correction factor for differences in quantum yields (Φ) and detection efficiencies (η)^33^ and has been found to be 0.9 for the FRET pair used^62^. Average intensity values of spectral crosstalk and direct excitation of acceptor fluorophores by the green laser were subtracted.

The resulting FRET efficiency histograms were fit to a sum of Gaussian distributions using Origin (OriginLabs). To quantify the fraction of native molecules during spontaneous folding, the area of the histogram corresponding to N-FLuc was divided by the total area of the histogram at each folding time point (0–10 h). This fraction was plotted against refolding time and then fitted to a single exponential function, yielding approximate rates of refolding. Data were recorded continuously over the 10 h period, then subdivided for analysis using a sliding window. The duration of the window was 30 min for early time points, and 1 h for later time points. Since folding continues during the measurement, time points are given as the midpoint of each recording interval, for example, the earliest time point (15 min) corresponds to data collected in the 0–30 min window. For FLucC, data for the first time point were summed over 1 h in order to include sufficient FRET events to generate a histogram that could be reliably fitted. The first time point for the spontaneous folding of FLucC is therefore marked as 30 min.

spFRET measurements at 37 °C were performed as described above, and the temperature was controlled using an Ibidi Heating System.

### Analytical ultracentrifugation (AUC)

To avoid aggregation, 200 nM FLuc-Atto532 (prepared as described above) was analysed in folding buffer with 5 M GuHCl. The DnaK-FLuc complex was prepared by dilution of GuHCl-denatured FLuc-Atto532 to 200 nM in folding buffer containing 3 µM DnaK, 1 µM DnaJ and 5 mM ATP. DnaK-FLuc complexes prepared under these conditions produced folded FLuc with a similar rate and yield to control reactions using un-labelled protein. Three independent replicates were performed for each condition.

Sedimentation velocity experiments were performed on an Optima XL-I analytical centrifuge (Beckman Inc., Indianapolis, In, U.S.A.) using an An-60 Ti rotor and double-sector 12 mm centrepieces. Buffer density was measured as 1.02054 kg L^−1^ using a DMA 5000 densitometer (Anton Paar, Graz, Austria). Protein concentration distribution was monitored at 532 nm, using 184,000 xg rotor speed. Time-derivative analysis was computed using the SEDFIT software package^65^, resulting in a c(s) distribution and an estimate for the molecular weight (from sedimentation coefficient and the diffusion coefficient, inferred from the peak width).

### Hydrogen/deuterium exchange-mass spectrometry (H/DX-MS)

For equilibrium H/DX-MS, native and DnaK-bound FLuc were prepared essentially as described above for the luminescence folding assays. N-FLuc was prepared at final concentration of 1 µM in HDX buffer (folding buffer with 5 mM ATP, and without 0.05% T20) and the DnaK-FLuc complex was prepared by dilution of GuHCl-denatured FLuc to final 1 µM in HDX buffer containing 10 µM DnaK and 3.3 µM DnaJ (residual GuHCl 50 mM). DnaK-FLuc complexes prepared under these conditions produced folded FLuc with a similar rate and yield to control reactions at lower concentrations of FLuc and chaperones described above. Three independent replicates were performed for each condition.

Deuterium exchange was initiated by 10-fold dilution into deuteration buffer (HDX buffer prepared in D_2_O), followed by incubation at 25 °C for 10 s, 30 s, 100 s or 300 s. DnaK-FLuc complexes were analysed immediately after they were prepared, to avoid release of FLuc over prolonged incubation times. Exchange reactions were quenched by addition of an equal volume of ice-cold quench buffer (100 mM sodium phosphate pH 2.1, 10 mM tris(2-carboxyethyl)phosphine (TCEP), 1 M GuHCl), to a final pH of 2.6.

For pulsed-label H/DX, DnaK-FLuc complexes were prepared as described above for the equilibrium H/DX experiments, and FLuc folding was initiated by addition of 5 µM GrpE. At different folding times (10 s, 5 min, 30 min), 5 µl aliquots of the reaction were withdrawn and added to 45 µl deuteration buffer. Deuterium labelling was allowed to proceed for 10 s, before quenching by addition of 50 µl quench buffer containing 20% glycerol, and snap-freezing in liquid N_2_. Samples were stored at −80 °C for no longer than one week before analysis.

Proteolysis, chromatography, peptide-mass analysis and data processing were performed as described previously^66^. To process spectra with bimodal patterns, all isotopes of the entire bimodal distribution were selected for processing, and relative deuterium incorporation was calculated based on the centroid of the isotopic distribution. Experiments were performed at least three times, and the error of mass measurement was <0.2 Da. All experiments were performed under identical conditions. Deuterium levels were therefore not corrected for back-exchange and all data are reported as relative. Sequence coverage was ~95% for N-FLuc, ~60% for FLuc in complex with DnaK, and ~60% for FLuc in the pulsed-label experiment.

The mass spectrometry proteomics data have been deposited to the ProteomeXchange Consortium via the PRIDE^67^ partner repository with the dataset identifier PXD016509.

### Computational prediction of DnaK binding sites on FLuc

DnaK binding sites on FLuc were predicted using the LIMBO web server^37^. Heptapeptide motifs with a score > 10 were considered to be high confidence DnaK binding sites. The sequence of *Photinus pyralis* luciferase (UniprotKB P08659) was used as an input.

### Reporting summary

Further information on research design is available in the Nature Research Reporting Summary linked to this article.

## Supplementary information

Supplementary Information

Reporting Summary

Description of Additional Supplementary Files

Supplementary Data 1

## Data Availability

All data presented in this study are available within the figures and in the Supplementary Information, including the Source Data file linked to this article. The source data underlying Fig. 2b, c, 3b–k, 4a–e, 6b, 7 and 8a, b and Supplementary Figs. 1a–e, 2a–h, 3a, b, 4a–c, 5a, b, 6, 8a–c, 9a, b and 10 are provided as a Source Data file. The search and raw files for the H/DX data are available via ProteomeXchange (PRIDE) with identifier PXD016509, and other data are available from the corresponding authors upon reasonable request.

## Source data

Source Data
