# Supplementary material for: Bacterial Hsp70 resolves misfolded states and accelerates productive folding of a multi-domain protein
Source: Nat Commun. 2020 Jan 17;11:365. doi: 10.1038/s41467-019-14245-4 (PMC6969021; doi:10.1038/s41467-019-14245-4)
Supplement: Supplementary file 1 — Supplementary Information [file 41467_2019_14245_MOESM1_ESM.pdf]

Supplementary Information

Bacterial Hsp70 resolves misfolded states and accelerates productive folding of a multi-domain protein

Imamoglu et al.

SUPPLEMENTARY FIGURES

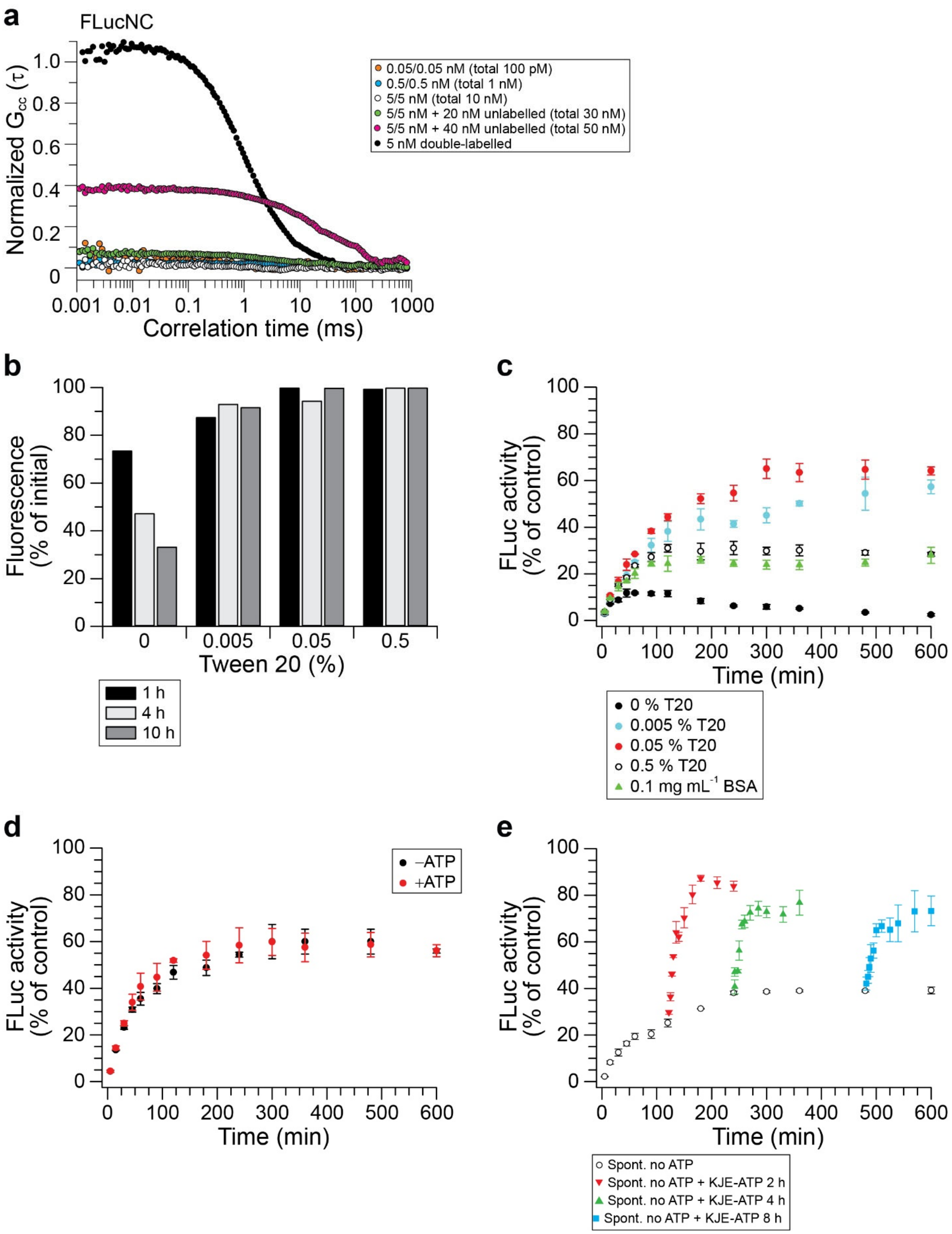

Supplementary Figure 1 | FLuc folding monitored by recovery of luminescence activity. a. FLuc is monomeric during refolding at concentrations <10 nM at 25 ºC. Equimolar mixtures of GuHCl-denatured FLuc labelled with Alexa532 or Alexa647 were diluted to total concentrations of 100 pM to 10 nM in folding buffer, and intermolecular association was monitored using dcFCCS. As a positive control mimicking a dimeric species, FLuc (5 nM) was labelled with both dyes simultaneously. Alternatively, FLuc aggregation was induced by increasing the total protein concentration to 30 or 50 nM by addition of unlabelled denatured FLuc. Samples were incubated at 25 ºC for 30 min before recording dcFCCS for 30 min. Representative measurements of 3 independent repeats are shown. **b**, 0.05 % T20 prevents tube adsorption during spontaneous folding of FLuc. Spontaneous refolding of GuHCl-denatured FLuc-Alexa647 was initiated by dilution to 1 nM in folding buffer supplemented with different amounts of T20. At the indicated time points, an aliquot was removed from the tube and recovered protein was quantified by fluorescence at 665 nm. The fluorescence at each time point was normalised to the initial fluorescence, recorded immediately after dilution into buffer. **c**, Spontaneous folding of FLuc at different concentrations of T20 and BSA. Refolding of GuHCl-denatured FLuc was initiated by dilution to 1 nM in folding buffer supplemented with T20 (0 – 0.5 %) or BSA (0.1 $mg.ml^{-1}$). Folding was assayed by recovery of luminescence activity as in Fig. 2b. **d**, ATP does not affect the rate or yield of spontaneous folding of FLuc. Refolding of GuHCl-denatured FLuc was initiated by dilution to 1 nM in folding buffer with or without 5 mM ATP, and assayed as in (d). **e**, Accelerated folding of FLuc by KJE is ATP-dependent. KJE-assisted folding of 1 nM FLuc was performed as in Fig. 2c, but in the absence of ATP. Reactions were supplemented with 5 mM ATP after 2, 4 or 8 h. Error bars represent s.d. (n=3). Source data are provided as a Source Data file.

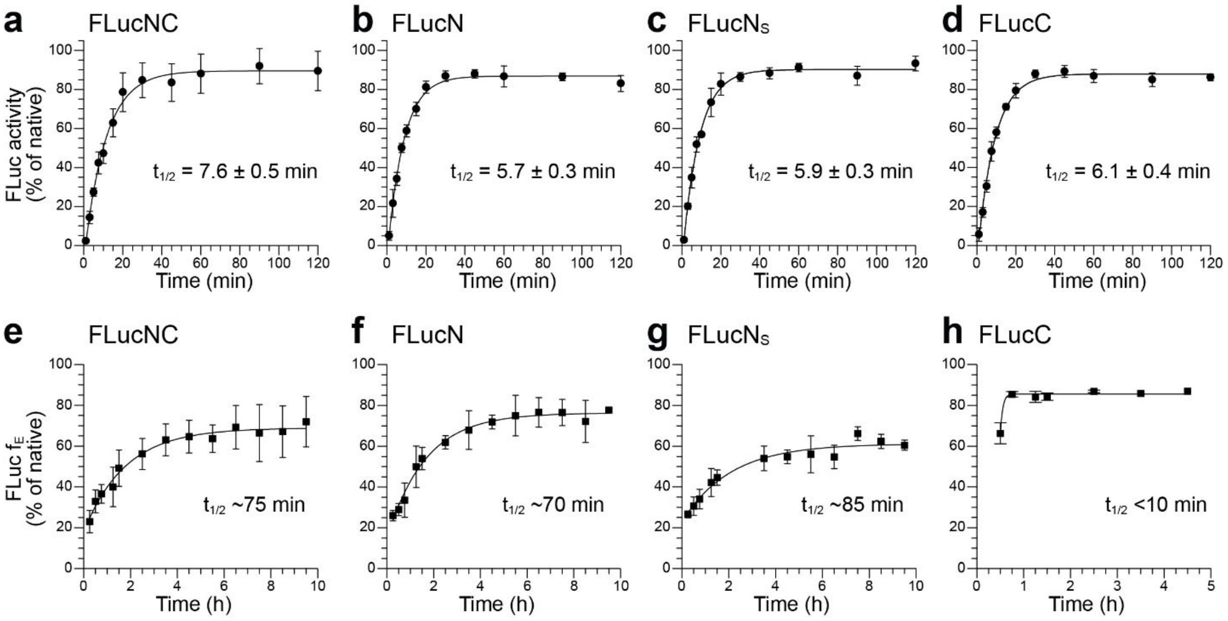

Supplementary Figure 2 | Spontaneous folding of FLuc monitored by spFRET. a-d, Fluorescent labelled FLuc proteins are competent to refold. FLucNC (**a**), FLucN (**b**), FLucN$_S$ (**c**) and FLucC (**d**) were diluted to 1 nM from 5 M GuHCl into folding buffer containing 0.3 µM DnaK, 0.1 µM DnaJ, 0.5 µM GrpE and 5 mM ATP. Folding at 25 ºC was monitored by luminesence assay. Error bars represent s.d. (n=4). **e-h**, Kinetics of spontaneous FLuc folding derived from spFRET experiments as in Fig. 3f-i. Conversion of the f_E distribution of denatured FLucNC (**e**), FLucN (**f**), FLucN$_S$ (**g**) and FLucC (**h**) towards the f_E of the native state was calculated by dividing the area of the f_E histogram corresponding to native protein by the total area of the f_E histogram at each folding time point (0-10 h). Time points correspond to the midpoint of each recording interval. Error bars represent s.d. (n=3). Source data are provided as a Source Data file.

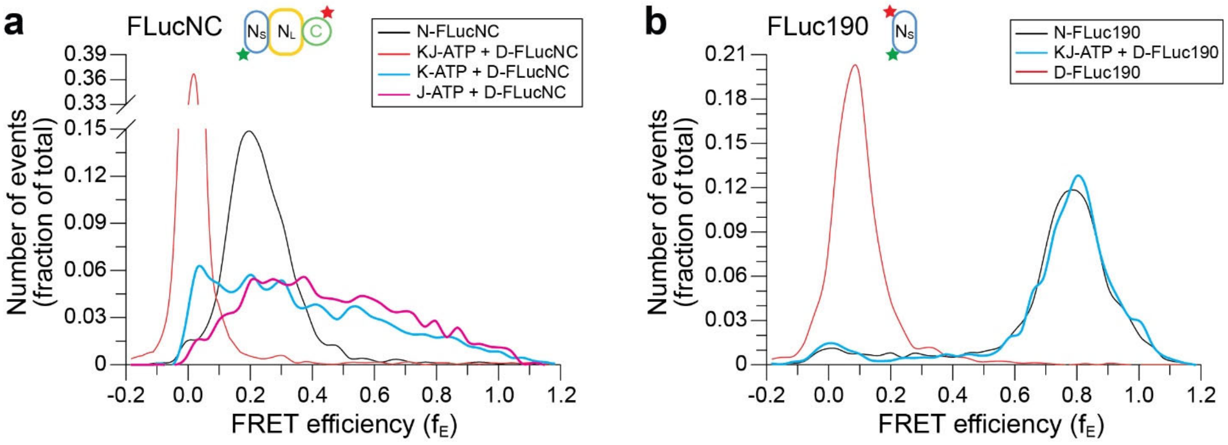

Supplementary Figure 3 | Chaperone-mediated unfolding of FLuc. a, Conformational expansion of FLuc requires both DnaK and DnaJ. GuHCl-denatured FLucNC (D-FLucNC) was diluted to 50 pM in folding buffer containing either 0.3 µM DnaK, 0.1 µM DnaJ or a mixture of both chaperones. ATP (5 mM) was always present. spFRET was recorded. Native FLucNC (N-FLucNC) is also shown for comparison. Representative measurements of 3 independent repeats are shown. **b,** FLuc190 escapes KJ-ATP binding due to rapid folding. FLuc190 was diluted from denaturant into folding buffer containing KJ-ATP as in (a), followed by spFRET analysis. N-FLuc190 and D-FLuc190 are shown for comparison. Representative measurements of 3 independent repeats are shown. spFRET data for each plot were recorded for 1 h and a minimum of 1000 events were collected. The f_E distribution of 50 pM native FLuc190 (N-FLuc190) is also shown. Source data are provided as a Source Data file.

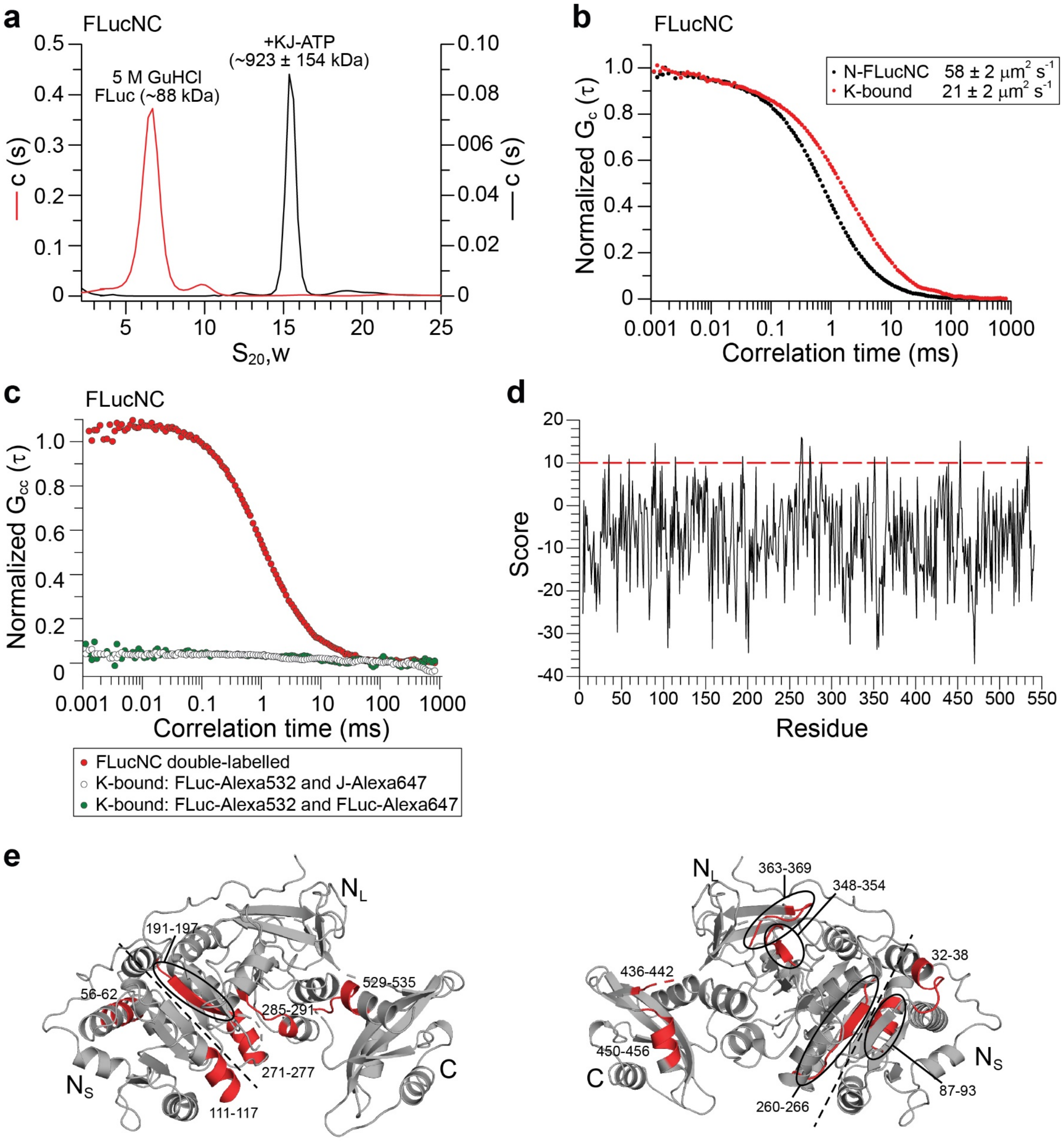

Supplementary Figure 4 | Characterization of chaperone-bound FLuc. a, Analytical ultracentrifugation and sedimentation velocity analysis of DnaK-bound FLuc. FLucNC (see Fig. 3a) labeled with Atto532 was unfolded in 5 M GuHCl and analysed either in 5 M GuHCl or upon dilution into folding buffer containing 3 μM DnaK, 1 μM DnaJ and 5 mM ATP. The final concentration of FLucNC was 200 nM. Shown in red is the sedimentation profile (absorption at 532 nm) of denatured FLucNC and in black of DnaK-bound FLucNC. X-axis is sedimentation coefficient and y-axis is sedimentation coefficient distribution. The apparent mass of complexes is indicated. Representative measurements of 3 independent repeats are shown. **b,** Fluorescence correlation spectroscopy (FCS) analysis of FLuc when bound to DnaK. Denatured FLucNC-

Atto532 (see Fig. 3a) was diluted to 50 pM in folding buffer containing 0.3 µM DnaK, 0.1 µM DnaJ and 5 mM ATP. The diffusion coefficients of native and DnaK-bound FLuc are indicated. Representative measurements of 3 independent repeats are shown. **c**, Chaperone-bound FLuc contains only one molecule of FLuc. Denatured wild-type FLuc labelled at the N-terminus with Alexa532 was diluted to 10 nM in folding buffer containing 0.3 µM DnaK, 90 nM DnaJ, 10 nM DnaJ-Alexa647 and 5 mM ATP. Alternatively, 50 pM denatured FLuc-Alexa532 was mixed with 50 pM denatured FLuc-Alexa647 and diluted into folding buffer containing 0.3 µM DnaK, 0.1 µM DnaJ and 5 mM ATP. FLucNC (5 nM; see Fig. 3a) double-labelled with Alexa532 and Alexa647 was analysed as a positive control. Representative measurements of 3 independent repeats are shown. Source data for Figs a-c are provided as a Source Data file. **d,** Predicted DnaK binding motifs in the sequence of FLuc analysed by LIMBO. Heptapeptide motifs with a score above 10 (red dashed line) are considered to be high confidence DnaK binders with a false discovery rate <1 %. **e,** Structure of FLuc with predicted DnaK interaction sites from (d) coloured red. The interface between the N_S and N_L subdomains is indicated with a dashed line.

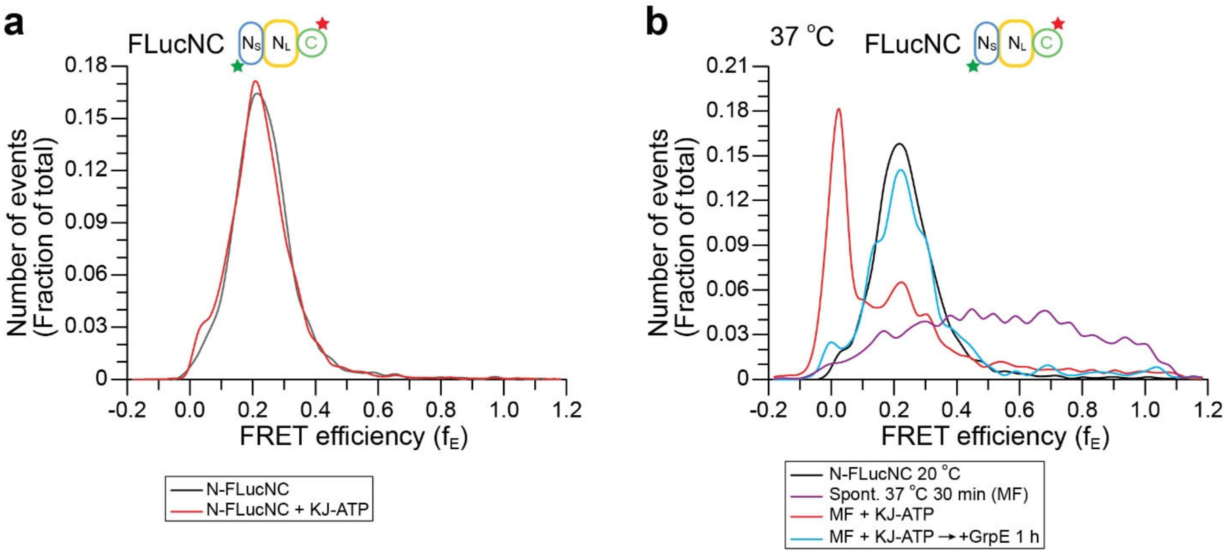

Supplementary Figure 5 | KJ-ATP recognize compact folding intermediates but not native FLuc. a, KJ-ATP do not bind N-FLuc. N-FLucNC was diluted to 50 pM in folding buffer with or without 0.3 µM DnaK, 0.1 µM DnaJ and 5 mM ATP and spFRET was recorded. Representative measurements of 3 independent repeats are shown. **b,** The KJE system rescues compact folding intermediates generated during spontaneous folding at 37 ºC. GuHCl-denatured FLucNC was diluted to 50 pM in folding buffer containing ATP (5 mM) and PBT (50 µM), followed by recording of spFRET data for 30 min. DnaK (0.3 µM) and DnaJ (0.1 µM) were added after 30 min to generate DnaK-bound FLuc (red), followed by GrpE (0.5 µM) to initiate folding (blue) for 1 h. spFRET data were recorded as in Fig. 4e. Representative measurements of 3 independent repeats are shown. Source data are provided as a Source Data file.

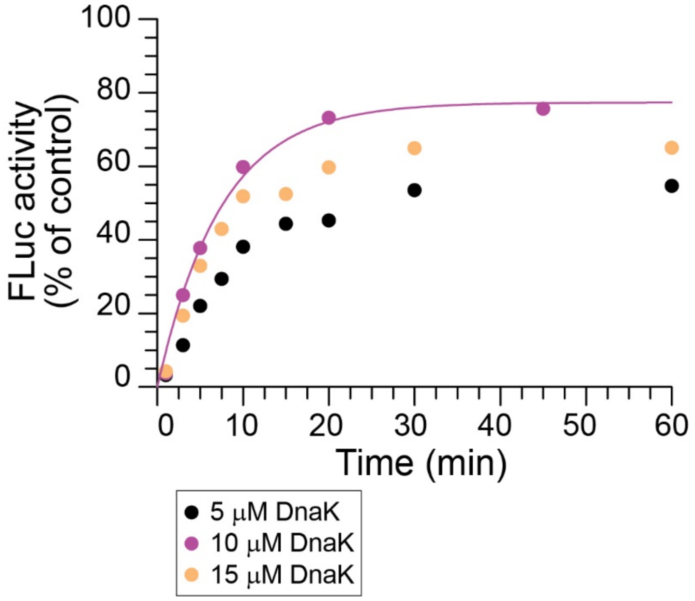

Supplementary Figure 6 | KJE-assisted folding of FLuc under conditions of H/DX-MS. KJE-assisted folding was analysed at a concentration of 1 µM FLuc. The concentration of DnaK varied from 5 to 15 µM, while maintaining a constant ratio between the chaperones of 3:1:1.5 (DnaK:DnaJ:GrpE). Folding was monitored by luminescence assay. H/DX measurements during FLuc refolding were performed at 10 µM DnaK/3.3 µM DnaJ/5 µM GrpE. Source data are provided as a Source Data file.

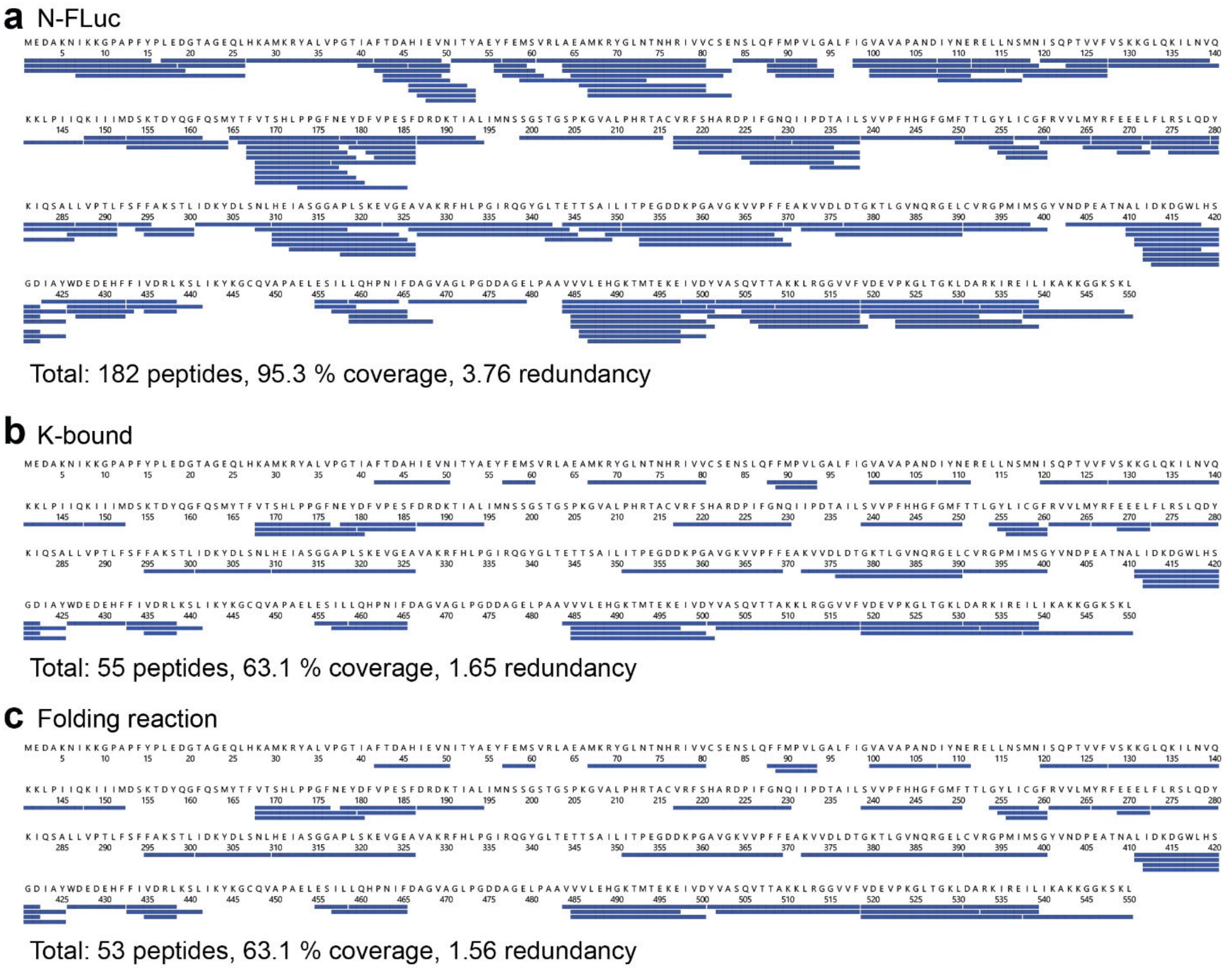

Supplementary Figure 7 | H/DX peptide coverage maps. a-c, Peptide coverage maps were generated in DynamX for N-FLuc (**a**), K-bound FLuc (**b**) and KJE-assisted folding of FLuc (**c**).

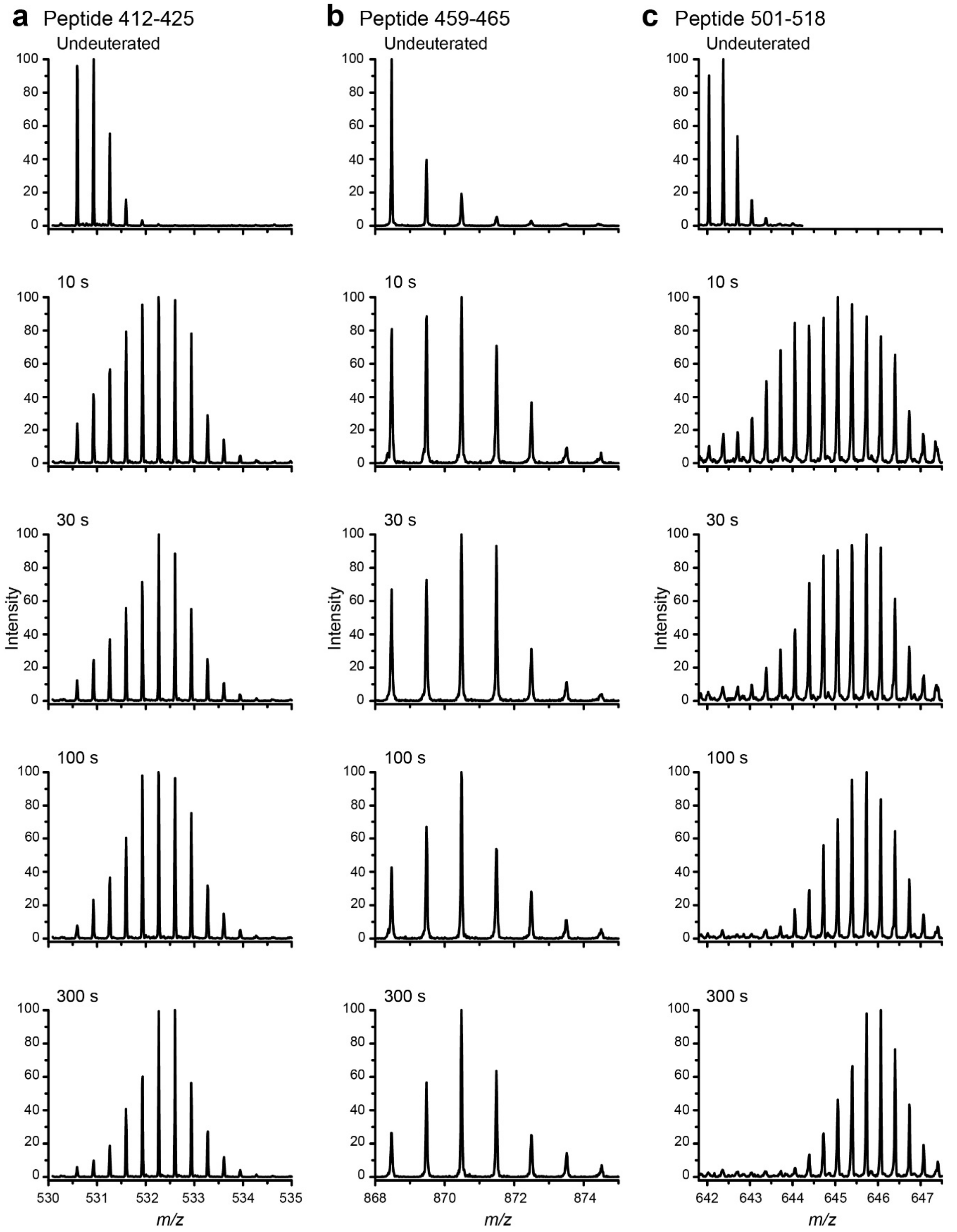

Supplementary Figure 8 | Bimodal peptide-mass distributions indicative of EX1 exchange in K-bound FLuc. a-c, Representative peptide mass spectra for peptide 412-425 (**a**), 459-465 (**b**) and 501-518 (**c**) in the undeuterated state, or after exposure to deuterium for 10, 30, 100 or 300 s. *m/z*, mass/charge. Source data are provided as a Source Data file.

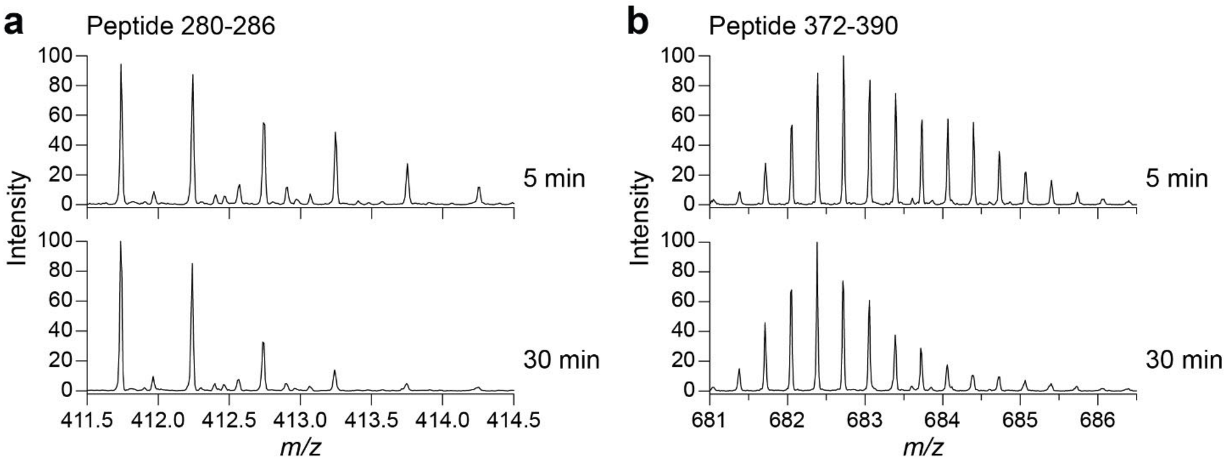

Supplementary Figure 9 | Bimodal peptide-mass distributions indicative of co-existing native and non-native populations during KJE-assisted folding. **a-b,** Representative peptide mass spectra for peptide 280-286 (**a**) and 372-390 (**b**) after 5 min or 30 min of assisted folding. Folding reactions were pulse-labelled with D_2O for 10 s at the indicated time points, and analysed as described in Fig. 6b and Methods. *m/z*, mass/charge. Source data are provided as a Source Data file.

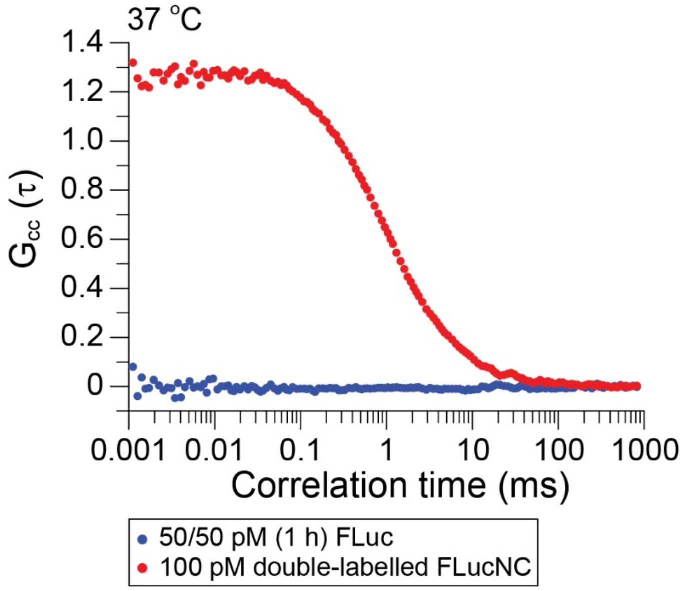

Supplementary Figure 10 | FLuc remains monomeric during spontaneous refolding at 100 pM and 37℃. An equimolar mixture of GuHCl-denatured FLuc labelled at the N-terminus with Alexa532 or Alexa647 was diluted to a total concentration of 100 pM in folding buffer at 37℃, and intermolecular association monitored using dcFCCS after 1 h of incubation. As a positive control mimicking a dimeric species, 100 pM double-labelled FLucNC was analysed. Representative measurements of 3 independent repeats are shown. Source data are provided as a Source Data file.

SUPPLEMENTARY TABLES

Assisted folding	FLuc (nM)	Temperature °C	DnaK (µM)	DnaJ (µM)	GrpE (µM)	Rate (min)	Yield (% of control)
	100	25	3	1	1.5	4.6 ± 0.3	89 ± 1
	0.1	25	0.3	0.1	0.5	4.5 ± 0.3	87 ± 2
	0.01	37	1	0.33	1.5	2.9 ± 0.3	86 ± 6
**Spontaneous folding**							
	200	25	-	-	-	50 ± 9	14 ± 1
	100	25	-	-	-	75 ± 7	23 ± 0.8
	50	25	-	-	-	65 ± 8	38 ± 2
	10	25	-	-	-	75 ± 6	56 ± 2
	1	25	-	-	-	75 ± 4	64 ± 2
	0.1	25	-	-	-	74 ± 4	61 ± 1
	0.01	37	-	-	-	38 ± 3	15 ± 0.4

Supplementary Table 1 | Rates and yields of KJE-assisted and spontaneous FLuc folding.

FLuc mutant	Primer	Sequence (5'- 3')
FLuc (D19C)	Forward	ATCCTCTAGAGTGTGGAACCGCTGGAG
	Reverse	CTCCAGCGGTTCCACACTCTAGAGGAT
FLuc (S170C)	Forward	CGTTCGTCACATGTCATCTACCTCC
	Reverse	GGAGGTAGATGACATGTGACGAACG
FLuc (E428C)	Forward	GCTTACTGGGACTGTGACGAACACTTC
	Reverse	GAAGTGTTCGTCACAGTCCCAGTAAGC
FLuc (D476C)	Forward	TCCCGACGATTGCGCCGGTGAACTTCC
	Reverse	GGAAGTTCACCGGCGCAATCGTCGGGA
FLuc (S504C)	Forward	GATTACGTCGCCTGTCAAGTAACAACC
	Reverse	GGTTGTTACTTGACAGGCGACGTAATC
FLuc190 (truncated)	Forward	CCACCACTAGACAATTGCACTGATAATGAATTC
	Reverse	TGATGATGATGTTTGTCACGATCAAAGGAC

Supplementary Table 2 | Primers used in this study.

Plasmid	Protein	Source
pET11d_ *DnaK*	DnaK	This study
pET11d_*DnaJ*	DnaJ	This study
pET3a_ *GrpE*	GrpE	This study
pET3a_ *FLuc*_FXa_Myc_6xHis	FLuc	This study
pET3a_ *FLuc*(D19C/S504C)_FXa_Myc_6xHis	FLucNC	This study
pET3a_ *FLuc*(D19C/S170C)_FXa_Myc_6xHis	FLucN$_S$	This study
pET3a_ *FLuc* (D19C/E428C)_FXa_Myc_6xHis	FLucN	This study
pET3a_ *FLuc*(D476C/S504C)_FXa_Myc_6xHis	FLucC	This study
pET3a_ *FLuc*(1-190)(D19C/S170C)_6xHis	FLuc190	This study

Supplementary Table 3 │ Plasmids, proteins and strains used or generated for this study.
