# Supplementary material for: Bacterial Hsp70 resolves misfolded states and accelerates productive folding of a multi-domain protein
Source: Nat Commun. 2020 Jan 17;11:365. doi: 10.1038/s41467-019-14245-4 (PMC6969021; doi:10.1038/s41467-019-14245-4)
Supplement: Supplementary file 3 — Description of Additional Supplementary Files [file 41467_2019_14245_MOESM3_ESM.pdf]

Description of Additional Supplementary Files

File name: Supplementary Data 1

Description: H/DX-MS data summary. **a,** Meta data analysis. n.a., not applicable. **b,** H/DX data continuous labelling. s.d., standard deviation. **c,** H/DX data pulse labelling. Peptides marked in bold showed a change in deuterium uptake of >1 Da during folding and were considered for further analysis. See Methods for further details. D, deuterium.
